# Cellular crosstalk and signaling networks in the rheumatoid arthritis synovial microenvironment

**DOI:** 10.1186/s12967-026-08482-7

**Published:** 2026-07-03

**Authors:** Maozhi Feng, Hongtai Chen, Lu Liao, Donghong Huang, Jun Shen, Lianbo Xiao, Qigui Lu, Pingjin Xie

**Affiliations:** 1 Shanghai University of Traditional Chinese Medicine, Shenzhen Hospital, 16 Xiantong N Rd, Shenzhen, 518001 China; 2 Guanghua Hospital, Affiliated to Shanghai University of Traditional Chinese Medicine, Shanghai University of Traditional Chinese Medicine, Shanghai, 200052 China

**Keywords:** Rheumatoid arthritis, Synovial microenvironment, Cellular crosstalk, Inflammatory activity, Remission, Signaling pathways

## Abstract

Rheumatoid arthritis (RA) is a chronic autoimmune disease characterized by synovial inflammation, pannus formation, and progressive cartilage and bone destruction. Within the RA synovial microenvironment, resident synoviocytes, mesenchymal stem cells, fibroblasts, adipocytes, vascular-associated cells, and diverse immune cell populations form a dynamic interaction network through direct contact and paracrine mediators, including cytokines, chemokines, complement components, and extracellular vesicles. This review summarizes how these cellular interactions drive RA along a pathological continuum from early autoimmune initiation, through middle-stage inflammatory amplification and synovial hyperplasia, to late fibrosis, pannus formation, dysregulated bone remodeling, and irreversible structural damage. Particular emphasis is placed on the dynamic balance between pathogenic cellular circuits and immunoregulatory programs within the synovial microenvironment, which helps determine whether the joint remains in an inflammatory-active state, re-enters a remission-associated and relatively rebalanced state, or progresses toward remission failure and structural injury. We further discuss the major signaling pathways that mediate these interactions, especially NF-κB, MAPK, JAK-STAT, TGF-β/Smad, and Wnt/β-catenin signaling, highlighting how pathway crosstalk contributes to inflammatory persistence, loss of tissue plasticity, and progressive remodeling. Importantly, because key cellular subsets and interaction programs may still retain partial plasticity during the early and middle stages of disease, stage-adapted modulation of these pathogenic networks may help restore synovial immune homeostasis, promote remission, delay disease progression, and reduce irreversible tissue damage. A deeper understanding of stage-specific cellular programs and interaction networks may therefore provide a stronger theoretical basis for mechanism-informed precision therapies in RA.

## Introduction

Rheumatoid arthritis (RA) is a major disabling autoimmune disease worldwide. RA pathogenesis is multifactorial and involves genetic, environmental, and immunological factors [1, 2]. The synovium serves as the primary site of RA pathology; in its normal state, it consists of a thin connective tissue layer composed of 1–2 cell layers, predominantly type A (macrophage-like) and type B (fibroblast-like) synoviocytes [3, 4]. As RA develops, the synovial tissue progressively transforms from an initially quiescent structure into a hyperplastic and inflammatory pannus-like lesion. This pathological process begins with early autoimmune initiation, progresses through an amplification phase characterized by persistent synovial inflammation and hyperplasia, before finally causing cartilage destruction, subchondral bone erosion, joint deformity, and functional impairment [5].

Over the past decades, advances in RA management, including conventional synthetic disease-modifying antirheumatic drugs (csDMARDs), biologic DMARDs, targeted synthetic DMARDs, and treat-to-target strategies, have substantially improved clinical outcomes and made sustained remission, or at least low disease activity, an achievable goal for many patients. However, therapeutic responses remain highly heterogeneous in clinical practice. A considerable proportion of patients still fail to achieve stable disease control despite sequential treatment with agents targeting different inflammatory pathways. This variability in treatment response suggests that current therapeutic paradigms, although effective for many patients, do not fully capture the biological complexity and heterogeneity of RA. Consequently, a deeper mechanistic understanding of the disease is still needed, particularly at the level of the synovial microenvironment, where local cellular interactions directly shape inflammatory persistence, tissue remodeling, and structural damage.

Recent single-cell sequencing and imaging mass cytometry studies have revealed marked cellular heterogeneity in the RA synovium, including resident synoviocytes, recruited mesenchymal stem cells (MSCs), fibroblasts, adipocytes, vascular endothelial cells, vascular smooth muscle cells (VSMCs), and immune cells including macrophages, T cells, B cells, and dendritic cells (DCs) [6]. These cells do not exist in isolation; they engage in extensive communication via direct contact or paracrine signaling to establish a complex network of cellular interactions. Aberrant interactions among these diverse cell types activate key signaling pathways that drive the sustained amplification of inflammation and pathological synovial hyperplasia, thereby functioning as a central engine of RA progression.

Importantly, the significance of these cellular networks extends beyond their role in promoting disease progression. Increasing evidence suggests that differences in cellular composition, activation state, and intercellular communication may also underlie divergent clinical outcomes, including sustained inflammatory activity, partial disease control, remission, and recurrent flare. Accordingly, RA should not be viewed solely as a disease of linear structural progression, but rather as a dynamic process shaped by continuous shifts in synovial cellular states and signaling programs. Understanding these microscopic changes is therefore essential not only for elucidating the core pathogenesis of RA, but also for explaining why current therapies produce variable outcomes across patients.

**Fig. 1:**
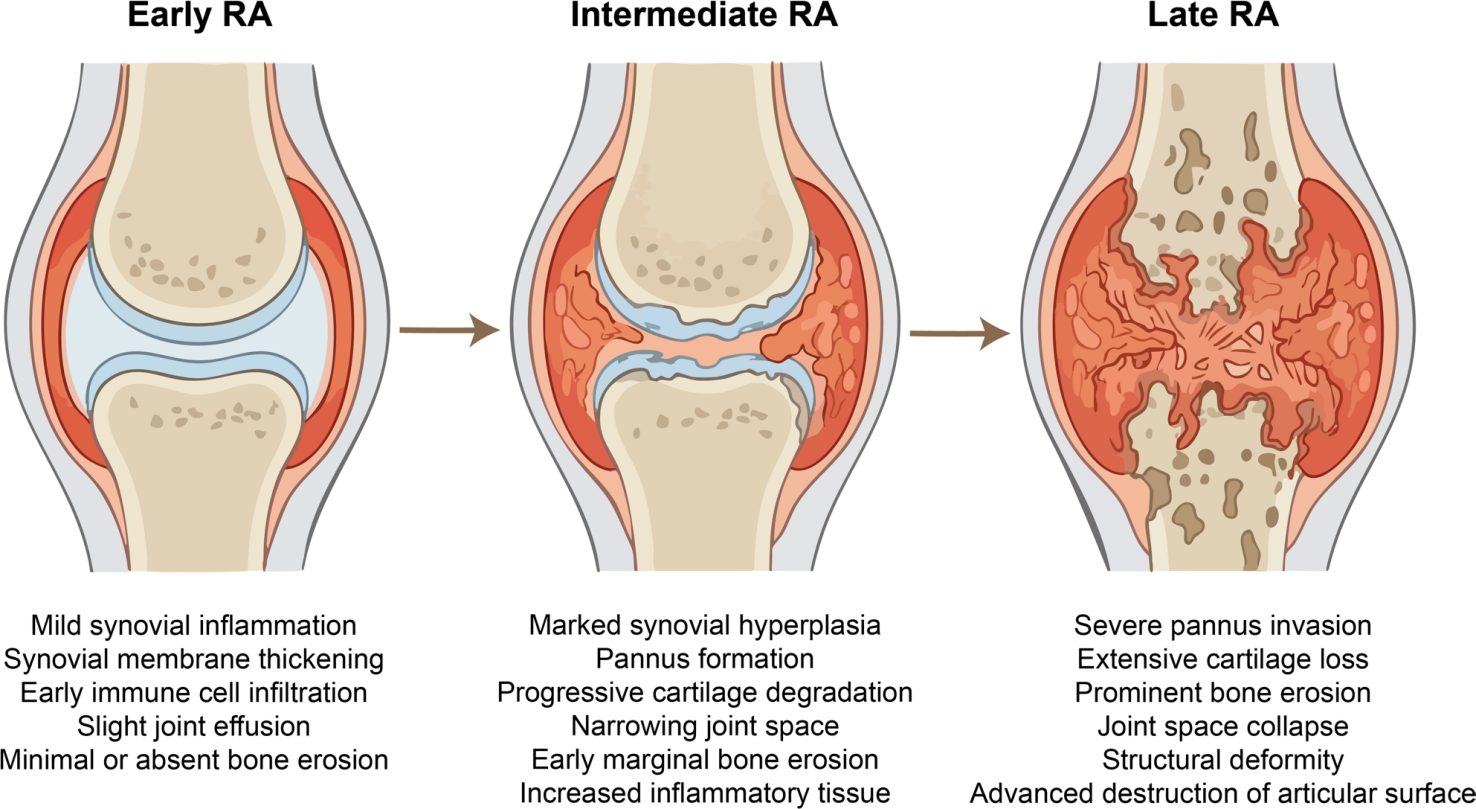
Stage-dependent pathological evolution of rheumatoid arthritis. This figure illustrates the pathological evolution of RA from early synovitis to late destructive joint remodeling. RA progresses along a pathological continuum from early immune-driven synovial inflammation, through an intermediate phase of inflammatory amplification and synovial hyperplasia, to late-stage pannus-mediated cartilage destruction, bone erosion, and joint deformity. This progression reflects the cumulative consequences of persistent inflammatory activity, aberrant stromal activation, and loss of local tissue homeostasis, thereby providing a morphological framework for understanding the role of synovial microenvironmental imbalance in disease progression and structural outcome. Abbreviations: RA, rheumatoid arthritis

Given this, a systematic analysis of synovial cellular crosstalk is of particular importance. Elucidating how key cell populations interact across different pathological stages and disease states may help clarify the mechanisms linking early immune activation, chronic synovial inflammation, and late structural damage. Critically, such insights may reveal therapeutic windows during the early and middle stages of RA, when critical cellular subsets and their functional states may still retain substantial plasticity. In this review, we summarize the major cellular components of the RA synovial microenvironment, discuss the principal interaction networks and associated signaling pathways that govern disease evolution, and highlight how modulation of these cellular programs may provide a theoretical basis for preventing the transition from reversible inflammation to irreversible structural damage. To guide the subsequent discussion, Fig. 1 illustrates the stage-dependent pathological evolution of RA, highlighting the transition from early immune-driven synovitis to intermediate inflammatory amplification and finally late pannus-mediated joint destruction.

## Core cell types and biological characteristics within the RA synovial microenvironment

**Fig. 2:**
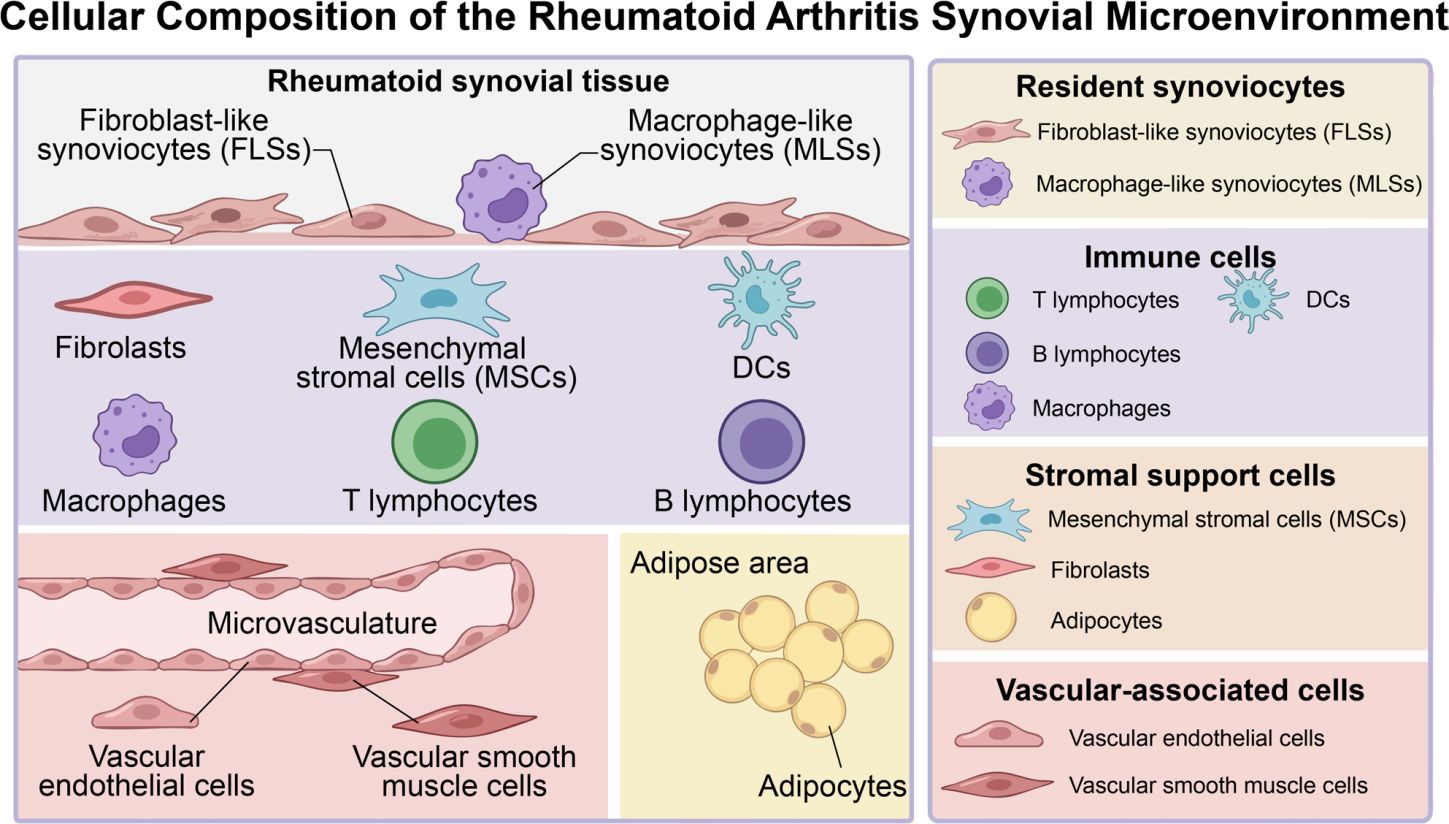
Cellular composition within the rheumatoid arthritis synovial microenvironment. This figure illustrates the major cellular components of the RA synovial microenvironment and their spatial organization within the synovial lining and sublining regions. The lining layer is mainly composed of type A synoviocytes/synovial tissue macrophages and type B synoviocytes/FLSs, whereas the sublining layer contains fibroblasts, MSCs, adipocytes, VECs, VSMCs, and infiltrating immune cells, including macrophages, DCs, T cells, and B cells. Abbreviations: RA, rheumatoid arthritis; FLSs, fibroblast-like synoviocytes; MSCs, mesenchymal stem cells; VECs, vascular endothelial cells; VSMCs, vascular smooth muscle cells; DCs, dendritic cells

The cellular composition of the RA synovial microenvironment is highly heterogeneous, with distinct cell types exhibiting significant differences in morphology, origin, and function; and these differences provide the structural and functional basis for intercellular interactions that collectively drive RA pathology. To provide a structural overview of the major stromal, vascular, metabolic, and immune cell populations discussed in this section, Fig. 2 summarizes the cellular composition of the RA synovial microenvironment and highlights their spatial distribution within the lining and sublining compartments.

### Resident synoviocytes

Resident synoviocytes, comprising type A and type B synoviocytes, are the primary constituents of the synovial lining layer. They maintain normal physiological function under homeostatic conditions but become aberrantly activated to drive inflammation in pathological states [7, 8].

Type A synoviocytes (macrophage-like synoviocytes, MLSs) are now commonly termed synovial tissue macrophages (STMs). In health, the synovial lining layer is dominated by embryonic-derived tissue-resident STMs [9, 10]. However, in inflammatory conditions such as RA, this resident population is expanded and functionally altered by the massive recruitment of monocyte-derived macrophages, which often acquire distinct pro-inflammatory phenotypes (pathological STMs) [11]. Their core biological characteristics encompass antigen presentation to T cells via MHC class I/II molecules, which initiates adaptive immune responses, and a potent secretory function that amplifies inflammation and tissue destruction through the release of pro-inflammatory cytokines (e.g., tumor necrosis factor-alpha [TNF-α], interleukin-1 beta [IL-1β], interleukin-6 [IL-6]), chemokines (e.g., monocyte chemoattractant protein-1 [MCP-1], c-x-c motif chemokine ligand 8 [CXCL8]), and matrix metalloproteinases (MMPs) [12–15].

Synovial fibroblasts (SFs) are the predominant resident mesenchymal population in the synovium. The lining-layer subset, commonly termed fibroblast-like synoviocytes (FLSs), constitutes 70–80% of the intima and is thought to derive from synovial mesenchymal progenitors or MSC-like populations [16]. Under homeostatic conditions, FLSs secrete synovial fluid components (e.g., hyaluronic acid [HA]) that maintain joint lubrication and cartilage nutrition [17, 18]. In RA, however, FLSs become aberrantly activated and acquire a tumor-like invasive phenotype with markedly altered biological properties: (1) enhanced proliferation with escape from normal growth control, leading to persistent synovial hyperplasia [19, 20]; (2) increased migration and invasion, enabling penetration of the cartilage matrix and direct participation in pannus formation [21, 22]; (3) dysregulated secretion of pro-inflammatory cytokines, chemokines, and MMPs (e.g., MMP-1, MMP-3, MMP-13), which amplify inflammation and cartilage degradation [23, 24]; and (4) aberrant immunomodulation, regulating immune cell activation and differentiation through the expression of co-stimulatory molecules and cytokine secretion [25]. Collectively, aberrant FLS activation is a central driver of synovial hyperplasia and tissue destruction in RA, and FLSs function as dynamic pathogenic effectors that connect early immune activation to middle-stage inflammatory amplification and synovial hyperplasia, and ultimately to late pannus formation and irreversible tissue damage.

### Mesenchymal-origin cells

The RA synovial interstitium contains diverse mesenchymal-origin cells, including MSCs, fibroblasts, and adipocytes, which undergo stage-dependent functional remodeling and collectively influence whether the synovial microenvironment remains relatively regulated, enters sustained inflammatory amplification, or progresses toward fibrosis and destructive remodeling. MSCs are adult stem cells with multilineage differentiation potential (e.g., into osteoblasts, chondrocytes, and adipocytes) and immunomodulatory capacity. Derived primarily from bone marrow, umbilical cord, and adipose tissue, they are recruited to inflamed synovial tissue through chemotactic signals within the RA microenvironment [26, 27]. Under homeostatic conditions, MSCs maintain immune homeostasis by secreting anti-inflammatory mediators (e.g., interleukin-10 [IL-10], transforming growth factor-beta [TGF-β]) and by regulating immune cell activity. In RA, high levels of pro-inflammatory cytokines (e.g., TNF-α and IL-1β) impair MSC differentiation and immunomodulatory capacity instead driving these cells toward a more pro-inflammatory or stromal-like phenotype, thereby promoting synovial hyperplasia and local inflammatory amplification [28–30].

Sublining fibroblasts reside in the interstitial region and are distinct from lining-layer subset FLSs in both origin and function [31]. Under homeostatic conditions, SFs primarily synthesize and remodel the extracellular matrix (ECM), thereby maintaining the structural integrity of synovial tissue [32, 33]. In RA, particularly during the late tissue destruction and remodeling stage, inflammatory stimulation activates these fibroblasts, resulting in enhanced proliferation and excessive ECM production (e.g., collagen and fibronectin). These activated fibroblasts also secrete pro-inflammatory cytokines and chemokines that cooperate with FLSs to promote synovial hyperplasia and fibrosis [34, 35].

Adipocytes are primarily located in the adjacent infrapatellar fat pad (IPFP) and interact with the synovium mainly at the tissue interface rather than by directly infiltrating the lining layer. Their principal function is the secretion of adipokines, including leptin, resistin, chemerin, and adiponectin [36, 37]. In RA, the adipokine secretion profile is altered, with increased levels of leptin and resistin, whereas the role of adiponectin appears to be complex and context-dependent. These changes modulate synovial and immune cell functions, thereby promoting inflammation; critically, they couple metabolic inflammation to stromal and immune-cell activation, reinforcing inflammatory activity, reducing the likelihood of durable remission, and facilitating progression toward chronic synovial pathology in RA [38–40].

### Vascular-associated cells

Angiogenesis is a hallmark of RA synovial hyperplasia, in which vascular-associated cells (vascular endothelial cells [VECs] and VSMCs) play central roles not only in inflammatory cell recruitment and nutrient delivery, but also in stabilizing middle-stage inflammatory activity and supporting the transition toward pannus expansion and late structural damage [41, 42]. The VECs form a single-cell layer lining the inner vascular wall and maintain vascular integrity and permeability under homeostatic conditions. In RA, angiogenic factors such as vascular endothelial growth factor (VEGF) and fibroblast growth factor 2 (FGF-2) secreted by FLSs and inflammatory cells activate VECs and promote their proliferation and migration, leading to the formation of new vascular networks (angiogenesis) [43]. Newly formed vessels provide nutrients and oxygen to proliferating synovial tissue and infiltrating inflammatory cells. These vessels also facilitate leukocyte recruitment through the expression of adhesion molecules, including vascular cell adhesion molecule 1 (VCAM-1) and intercellular adhesion molecule 1 (ICAM-1), thereby amplifying the inflammatory response [44, 45].

The VSMCs reside in the tunica media and primarily maintain vascular wall tone and structural stability [46]. In RA, inflammatory and angiogenic signals activate VSMCs, resulting in enhanced proliferation and migration that contribute to new vessel formation and vascular remodeling. These activated VSMCs additionally secrete pro-inflammatory cytokines and vasoactive mediators (e.g., angiotensin II [Ang II]), which further regulate the function of VECs and angiogenesis and exacerbate synovial microenvironmental dysregulation [47].

### Immune cells

Immune cell infiltration and functional dysregulation are hallmarks of RA, and within this context, diverse immune populations interact with synoviocytes to initiate and sustain autoimmune responses [48]. Macrophages are among the most abundant infiltrating immune cells in synovial tissue. In addition to resident type A synoviocytes, this population includes monocyte-derived macrophages recruited from peripheral blood [49]. In RA, synovial macrophages exhibit a polarization imbalance characterized by predominance of M1-like pro-inflammatory programs and insufficient M2-like inflammation-resolving activity. M1-like macrophages promote tissue damage by releasing pro-inflammatory cytokines (TNF-α, IL-1β, and IL-6), chemokines, and MMPs, while also enhancing antigen presentation and T-cell activation [50, 51]. By contrast, M2-like macrophages contribute to immune regulation and inflammation resolution, and their relative insufficiency or functional impairment may limit restoration of synovial homeostasis. Therefore, disruption of the M1/M2 balance constitutes an important cellular basis for persistent inflammatory activity in RA.

T lymphocytes are central regulators of the adaptive immune response in RA and include CD4⁺ and CD8⁺ subsets. Dysregulation of CD4⁺ T cell subsets, particularly the imbalance between T helper 17 (Th17) cells and regulatory T (Treg) cells, represents a central mechanism of immune dysregulation in RA [52, 53]. Th17 cells secrete pro-inflammatory cytokines such as interleukin-17 A (IL-17 A), interleukin-17 F (IL-17 F), and interleukin-22 (IL-22), which activate synoviocytes and other immune cells, amplify inflammatory responses, and contribute to tissue destruction. By contrast, Treg cells maintain immune tolerance and suppress excessive inflammation mainly through anti-inflammatory mediators such as IL-10 and TGF-β. In RA, however, the Th17/Treg balance is skewed toward Th17-dominant inflammatory responses, driven in part by cytokine milieus rich in IL-6 and IL-23, while Treg cells may be numerically reduced, functionally impaired, or destabilized, thereby limiting their capacity to restrain aberrant immune activation [54].

B lymphocytes contribute to RA pathogenesis through multiple mechanisms. They produce autoantibodies (e.g., rheumatoid factor [RF]; anti-cyclic citrullinated peptide [anti-CCP] antibodies) that form immune complexes, activate the complement, and exacerbate inflammation; function as antigen-presenting cells to stimulate T cell responses; and secrete cytokines that shape the inflammatory microenvironment [55–57]. During the early autoimmune initiation stage, DCs, the most potent antigen-presenting cells, trigger the RA immune response by activating naïve T cells and directing T cell subset differentiation [58, 59]. Collectively, these immune and stromal cell subsets not only participate in RA pathogenesis, but also define the dynamic balance between pathogenic and immunoregulatory programs within the synovial microenvironment. During the early phase of disease, DC-driven antigen presentation, macrophage activation, and Th17-skewed T-cell responses help initiate and consolidate autoimmunity. As RA progresses, sustained predominance of M1-like macrophages, Th17 cells, activated FLSs, and pro-inflammatory stromal responses amplifies synovial inflammation and promotes hyperplastic remodeling. By contrast, M2-like macrophages, Treg cells, and immunomodulatory MSCs constitute key components of the regulatory network that restrains pathogenic inflammation and supports remission-associated states. Therefore, failure to re-establish equilibrium between these opposing cellular programs may represent an important mechanistic basis for persistent inflammatory activity, remission failure, and subsequent structural progression in RA.

## Core interaction modes among RA synovial cells

Within the RA synovial microenvironment, diverse cell types communicate through direct contact or paracrine signaling, forming positive feedback loops that sustain inflammation, drive synovial hyperplasia, and promote tissue destruction. Collectively, these interactions constitute a central regulatory network underlying RA pathology.

### Interactions between resident synoviocytes: bidirectional synergistic activation of type A and type B synoviocytes

**Fig. 3:**
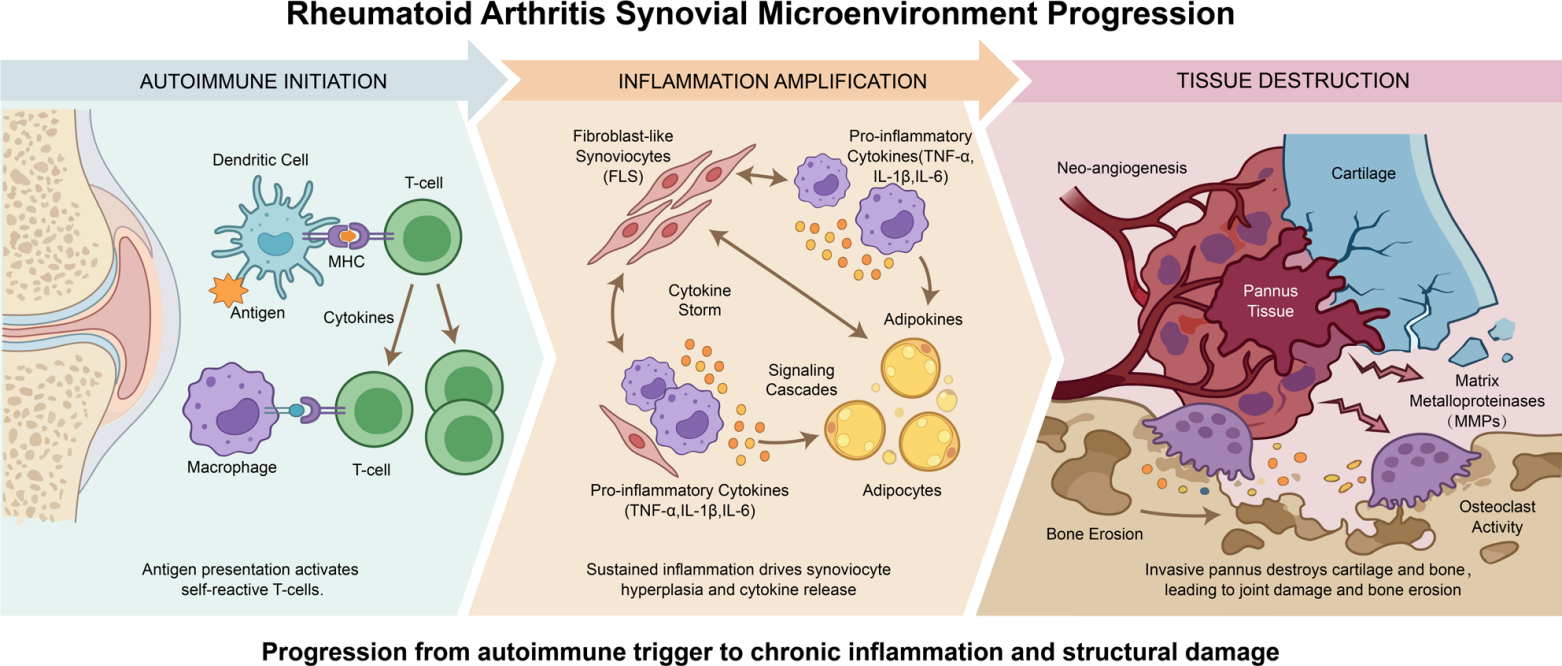
Stage-wise progression of cellular interactions in the RA synovial microenvironment. This figure illustrates the stage-wise progression of cellular interactions in the RA synovial microenvironment, from autoimmune initiation to inflammatory amplification and ultimately tissue destruction. In the early phase, antigen-presenting cells and macrophages activate autoreactive T cells and initiate local inflammatory responses. In the intermediate phase, fibroblast-like synoviocytes (FLSs), macrophages, and adipocytes engage in reciprocal cytokine-mediated interactions that amplify synovial inflammation and promote synoviocyte hyperplasia. In the late phase, neo-angiogenesis, pannus formation, matrix-degrading enzymes, and osteoclast activation cooperatively drive cartilage destruction and bone erosion. Together, these stage-dependent cellular interactions illustrate how the RA synovial microenvironment evolves from immune activation to chronic inflammation and structural joint damage. Abbreviations: RA, rheumatoid arthritis; FLSs, fibroblast-like synoviocytes

To contextualize the interactions discussed below, Fig. 3 provides a stage-oriented overview of how cellular interactions in the RA synovial microenvironment evolve from autoimmune initiation to inflammatory amplification and, ultimately, tissue-destructive remodeling.

Among these axes, the bidirectional crosstalk between type A and type B synoviocytes represents one of the earliest and most important amplifiers of synovial inflammation. Particularly during the transition from early immune activation to inflammatory amplification and synovial hyperplasia, these two synoviocyte populations form self-reinforcing loops through paracrine signaling and direct cell contact, thereby promoting persistent inflammation and setting the stage for subsequent tissue damage [60, 61]. Upon activation by inflammatory stimuli (e.g., lipopolysaccharides and immune complexes) during the early autoimmune initiation phase, type A synoviocytes release pro-inflammatory cytokines such as TNF-α and IL-1β. These cytokines bind to their receptors on FLSs, inducing a pro-inflammatory phenotype characterized by the production of IL-6, interleukin-8 (IL-8), and MMP-3. This phenotypic shift not only exacerbates persistent synovial inflammation but also pushes the microenvironment toward the subsequent tissue destruction stage by triggering early cartilage damage [62–64]. The IL-6 derived from both FLSs and type A synoviocytes further activates the intracellular Janus kinase/signal transducer and activator of transcription (JAK-STAT) pathway in an autocrine manner, reinforcing inflammatory cytokine production [65].

Activated FLSs additionally secrete complement component C3, which is cleaved to generate C3a. C3a binds to C3aR on type A synoviocytes and induces IFN-β secretion, which in turn enhances IL-6 and MMP-1/3 production by FLSs and promotes their invasive capacity. Consistently, in a collagen-induced arthritis (CIA) mouse model, blockade of the C3a-C3aR signaling pathway disrupted communication between type A synoviocytes and FLSs and significantly reduced synovial inflammation and joint destruction, underscoring the importance of this interaction in RA pathology [66]. Type A synoviocytes and FLSs also interact through direct physical contact. For example, ICAM-1 on FLSs binds to macrophage antigen-1 (Mac-1, or CD11b/CD18) on type A synoviocytes, enhancing cellular adhesion and facilitating direct inflammatory signaling [67, 68].

### Interactions between synoviocytes and mesenchymal stem cells: immune regulation and differentiation balance

After recruitment to the RA synovium, exogenous or endogenous MSCs communicate closely with FLSs and type A synoviocytes through paracrine signaling and direct cell contact. During the early stages of disease, these bidirectional interactions influence both inflammation and tissue repair [69]. In the early stages, MSCs attempt to maintain homeostasis; however, as the disease progresses into the inflammation amplification stage, this balance is disrupted. MSCs exert immunomodulatory effects and can inhibit aberrant synoviocyte activation through paracrine mechanisms. For example, human umbilical cord-derived MSCs secrete anti-inflammatory mediators such as IL-10 and tumor necrosis factor-stimulated gene 6 (TSG-6), which inhibit nuclear factor kappa-light-chain-enhancer of activated B cells (NF-κB) signaling in FLSs. This suppression downregulates invasion-associated enzymes (e.g., MMP-1 and MMP-9) and reduces FLS proliferation, migration, and invasion. MSCs also secrete indoleamine 2,3-dioxygenase (IDO), prostaglandin E2 (PGE2), and TSG-6, which promote polarization of type A synoviocytes toward an anti-inflammatory M2 phenotype. This shift reduces pro-inflammatory cytokine release and antigen presentation, thereby alleviating synovial inflammation [70, 71].

Conversely, high levels of inflammatory signals in RA also regulate MSC function and differentiation [72]. Activated FLSs and macrophages release TNF-α and IL-1β, which activate NF-κB signaling in MSCs and downregulate key transcription factors such as runt-related transcription factor 2 (RUNX2). This inhibition impairs osteogenic differentiation and promotes differentiation arrest and cellular senescence [73]. Sustained exposure to inflammatory cytokines (e.g., TNF-α and IL-1β) can also drive MSCs toward a pro-inflammatory MSC1 phenotype or a senescence-associated secretory phenotype (SASP). These altered MSCs secrete pro-inflammatory mediators such as IL-6, IL-8, and c-c motif chemokine ligand 2 (CCL2), which recruit immune cells and promote Th17 cell differentiation. This creates a positive feedback loop that further exacerbates synovial inflammation [74]. Disruption of the synoviocyte-MSC interaction balance, characterized by reduced immunosuppressive capacity and enhanced pro-inflammatory activity, contributes to the chronicity of RA inflammation and firmly establishes the inflammatory amplification phase. MSCs communicate with synoviocytes through direct contact mediated by gap junction proteins (e.g., Connexin 43) or adhesion molecules (e.g., CD44, ICAM-1), which may regulate cellular metabolism and proliferation. However, the precise contact-dependent mechanisms remain to be clarified [75].

Taken together, the synoviocyte–MSC axis may function as an important determinant of whether the synovial microenvironment remains relatively controlled or shifts toward persistent inflammatory activity. In the early stage, MSC-mediated immunoregulation may help counterbalance synovial inflammation; however, as inflammatory signals accumulate, disruption of this regulatory axis promotes the transition toward sustained inflammatory amplification. Additionally, disruption of the synoviocyte–MSC axis may serve as an important mechanistic link between sustained inflammatory amplification and late-stage structural damage through dysregulated bone remodeling. Persistent inflammatory stimulation not only weakens the immunoregulatory capacity of MSCs, but also suppresses their osteogenic differentiation potential, thereby limiting reparative bone formation within the diseased joint. Consequently, chronic crosstalk among activated synoviocytes, macrophages, and Th17 cells may further establish a microenvironment that favors osteoclast-mediated bone resorption while weakening osteoblast-associated repair capacity. Thus, impaired osteogenic repair and enhanced bone resorption together constitute an important mechanism by which persistent synovial inflammation progresses toward subchondral bone erosion and irreversible joint destruction.

### Interactions between synoviocytes and fibroblasts: synergistic promotion of synovial fibrosis and hyperplasia

The tripartite synergistic interaction among type A synoviocytes, FLSs, and sublining fibroblasts constitutes a central mechanism underlying pathological synovial remodeling during the inflammation amplification and tissue destruction stages. This network primarily orchestrates aberrant ECM remodeling and fibrosis, in which activated type A synoviocytes initiate an inflammatory cascade that stimulates sublining fibroblasts and lining FLSs to secrete abundant cytokines and matrix components, thereby establishing a pro-inflammatory and pro-fibrotic positive feedback loop that exacerbates synovial hyperplasia and directly drives the formation of the destructive pannus tissue [76, 77]. Under homeostatic homeostasis, SFs (particularly lining FLSs) remain largely quiescent, synthesizing HA and lubricin (Prg4) for joint lubrication while maintaining synovial structural integrity by balancing the synthesis and degradation of ECM components such as collagen [78, 79]. In RA, key mediators such as TGF-β and TNF-α secreted by type A (macrophage-like) synoviocytes potently activate sublining fibroblasts and induce a phenotypic transition characterized by enhanced proliferation and excessive ECM synthesis. These activated fibroblasts secrete substantial amounts of collagen (types I and III), fibronectin, and laminin, resulting in excessive extracellular deposition and consequent synovial fibrosis [61, 76].

Activated sublining fibroblasts reciprocally activate lining FLSs by secreting high levels of key pro-inflammatory mediators such as IL-6 and CXCL8, thereby significantly enhancing the proliferation and invasive potential of FLSs. This interaction establishes an “inflammation amplification-tissue destruction” positive feedback loop between fibroblast subsets in distinct synovial regions, which serves as a key mechanism driving irreversible joint structural damage [80]. Furthermore, these cells reinforce synergistic effects via cell-extracellular matrix (cell-ECM) interactions. For example, high expression of integrin α5β1 on fibroblasts enables specific binding to fibronectin-enriched microenvironments, activating the focal adhesion kinase (FAK)-phosphoinositide 3-kinase (PI3K)-Akt signaling pathway to confer anoikis resistance and promote directional migration and invasion, thereby collectively pushing the joint into the irreversible tissue destruction phase [81, 82]. The degree of synovial fibrosis correlates closely with joint deformity in RA patients, and inhibition of the FLS-fibroblast interaction significantly attenuates fibrosis and joint destruction, which highlights this interaction as a critical regulatory node in RA tissue injury.

### Interactions between synoviocytes and adipocytes: adipokine-mediated inflammation amplification

Adipocytes located at the synovial periphery engage in close interactions with FLSs and type A synoviocytes, serving as a critical metabolic and inflammatory engine during the inflammation amplification stage [83, 84]. The resulting adipose-synovial crosstalk provides synoviocytes with sustained inflammatory signals and metabolic substrates, thereby amplifying inflammation and accelerating the progression toward joint destruction in RA. Studies have demonstrated significantly elevated levels of pro-inflammatory adipokines, particularly leptin, resistin, and chemerin, in the synovial tissue and fluid of patients with RA [85, 86]. These factors bind to specific receptors on synoviocytes (e.g., leptin receptor [Ob-R], adenylate cyclase-associated protein 1 [CAP1], chemerin chemokine-like receptor 1 [ChemR23]), activating downstream mitogen-activated protein kinase (MAPK), PI3K/Akt, and NF-κB signaling cascades, and thereby inducing robust secretion of IL-6, IL-8, and MMPs by FLSs, which enhances their pro-inflammatory and invasive activities [87, 88].

For instance, leptin binds to the long-form receptor (Ob-Rb) on FLSs, inducing receptor dimerization and phosphorylation and subsequently activating the Janus kinase 2 (JAK2)-signal transducer and activator of transcription 3 (STAT3) signaling pathway. Activated STAT3 translocates to the nucleus to promote the transcription of Cyclin D1 and MMP-13, thereby enhancing the aberrant proliferation and cartilage-invasive capacity of FLSs. Additionally, leptin enhances the antigen-presenting function of type A synoviocytes, promoting T-cell activation and amplifying the adaptive immune response. Resistin binds to CAP1 or toll-like receptor 4 (TLR4) on FLSs, inducing inhibitor of nuclear factor kappa-B alpha (IκBα) phosphorylation and degradation, thereby triggering NF-κB nuclear translocation and activation. This process initiates transcription of downstream target genes, driving robust secretion of key pro-inflammatory cytokines such as IL-6 and TNF-α from FLSs and establishing an inflammatory cascade that exacerbates synovial pathology [89–91]. Chemerin promotes FLS migration and invasion primarily through ChemR23 (CMKLR1)-dependent signaling and also recruits immune cells such as monocytes and T cells into synovial tissue [92, 93].

Conversely, high levels of TNF-α and IL-6 secreted by activated synoviocytes (particularly type A) act as retrograde regulatory signals on adjacent adipocytes. These cytokines significantly downregulate the synthesis of the anti-inflammatory adipokine adiponectin by inhibiting the expression and activity of the transcription factor peroxisome proliferator-activated receptor gamma (PPARγ). Simultaneously, they activate the NF-κB and STAT3 pathways, inducing adipocytes to release additional leptin, IL-6, and MCP-1. This process drives the pro-inflammatory reprogramming of adipose tissue, locking the joint in a maladaptive synovial inflammation-adipose dysfunction positive feedback loop that sustains inflammatory activity, increases the stability of pathogenic inflammation, and reduces the capacity of the synovial microenvironment to re-enter a remission-associated state. Furthermore, adipocyte interactions with immune cells (e.g., macrophages and T cells) indirectly modulate synovial inflammation. For example, adipose tissue macrophages activated by adipokines secrete abundant pro-inflammatory cytokines that further regulate synoviocyte function, thereby creating a multi-level adipocyte-immune cell-synoviocyte inflammation-amplification loop.

### Interactions between synoviocytes and vascular-associated cells: driving angiogenesis and synovial hyperplasia

As the disease advances to the pannus formation and tissue destruction stage, interactions between VECs and VSMCs become major drivers of RA angiogenesis, forming a synergistic regulatory loop that not only supports synovial hyperplasia and inflammatory infiltration, but also creates a permissive niche for continued immune-cell trafficking and mediator exchange, thereby making inflammatory activity more difficult to extinguish and favoring progression toward irreversible structural injury [94]. On one hand, activated FLSs and type A synoviocytes secrete abundant angiogenic factors, including VEGF, FGF-2, and platelet-derived growth factor (PDGF), which bind to receptors on VECs and stimulate proliferation, migration, and lumen formation, thereby promoting neovascularization. Additionally, FLS-secreted MMPs degrade ECM components (e.g., collagen and fibronectin), mechanistically facilitating the destructive expansion of the pannus and creating space for VEC migration and neovascular extension [95]. On the other hand, activated VECs reciprocally regulate synoviocyte and inflammatory cell functions by secreting cytokines and expressing adhesion molecules [96]. For example, VEC-secreted PDGF and TGF-β promote proliferation of FLSs and fibroblasts while enhancing their pro-inflammatory activity [97, 98]. VCAM-1 and ICAM-1 expressed on VECs bind to integrins (e.g., very late antigen-4 [α4β1] and lymphocyte function-associated antigen-1 [LFA-1]) on immune cells, mediating their infiltration into synovial tissue and thereby amplifying inflammation [99].

In addition, VSMCs regulate neovascular maturity and stability through close physical contact and paracrine crosstalk with VECs [100]. Activated by the RA microenvironment, VSMCs both secrete Ang II through the local renin-angiotensin system (RAS) and significantly upregulate VEGF expression, which together enhance VEC proliferation and lumen sprouting [101]. Additionally, VSMCs secrete angiopoietin-1 (Ang-1), which binds to Tie2 (TEK) receptors on VECs to maintain vascular wall stability and remodeling [102]. However, in RA, this homeostatic mechanism is frequently dysregulated, resulting in neovessels with disorganized structure and increased permeability. Furthermore, VSMCs promote FLS proliferation and invasion through direct interactions, synergistically exacerbating synovial hyperplasia and pannus formation [103]. Recent studies indicate that FLS-derived exosomes can encapsulate angiogenic factors such as VEGF and deliver them to VECs to modulate their function and enhance angiogenesis, representing a novel regulatory dimension of synoviocyte-vascular cell interactions [104].

### Interactions between synoviocytes and immune cells: dysregulated immune responses and inflammation amplification

The interactions between synoviocytes (particularly FLSs) and immune cells constitute a central mechanism underlying immune dysregulation in RA and act as a major driver during the autoimmune initiation phase. FLSs act as inflammatory effector cells and may undergo phenotypic reprogramming characterized by inducible upregulation of major histocompatibility complex class II (MHC class II; HLA-DR). This change may confer partial antigen-presenting features, thereby enabling FLSs to modulate CD4⁺ T-cell responses under inflammatory conditions. Furthermore, FLSs secrete c-x-c motif chemokine ligand 13 (CXCL13) and B-cell activating factor (BAFF) to recruit and sustain peripheral helper T cells (Tph cells) and B cells, thereby promoting the formation of ectopic lymphoid structures (ELS). This multidimensional intercellular crosstalk establishes a self-amplifying feedforward loop that sustains chronic inflammation and promotes persistent loss of self-tolerance [105, 106] as the disease transitions into the robust inflammation amplification stage.

#### Interactions between synoviocytes and macrophages/DCs

As pivotal antigen-presenting cells, macrophages and DCs interact with synoviocytes to initiate the earliest autoimmune responses and subsequently amplify inflammatory signaling. On one hand, FLSs may acquire partial antigen-presenting features in inflammatory environments, including inducible HLA-DR expression; however, their ability to provide full co-stimulatory signaling appears limited and context-dependent [107]. Through these mechanisms, FLSs may modulate CD4⁺ T-cell activation, although their capacity to directly induce full clonal expansion remains incompletely defined [108]. Beyond antigen presentation, FLSs activate macrophages and DCs in an antigen-independent manner by secreting granulocyte-macrophage colony-stimulating factor (GM-CSF) and expressing ICAM-1 and VCAM-1. GM-CSF acts as a critical survival factor that inhibits synovial macrophage apoptosis, while contact-dependent signals synergistically promote macrophage polarization toward a pro-inflammatory phenotype, thereby establishing a local fibroblast-macrophage inflammatory amplification loop [109]. Additionally, FLSs induce macrophage polarization toward the M1 pro-inflammatory phenotype through GM-CSF and IL-6 secretion, enhancing antigen presentation and pro-inflammatory cytokine (TNF-α and IL-1β) production [110]. Conversely, activated macrophages and DCs reciprocally activate synoviocytes by secreting pro-inflammatory cytokines and chemokines. For instance, macrophage-derived TNF-α and IL-1β markedly enhance the pro-inflammatory functions of FLSs [34]. Similarly, interleukin-12 (IL-12) and interleukin-23 (IL-23) secreted by DCs drive T cell-mediated immune responses and also directly or indirectly modulate the pathogenic behavior of FLSs, further exacerbating synovial inflammation [111]. Furthermore, macrophages and type A synoviocytes may contribute to local complement activation through the production of components such as C3 and C5, thereby generating complement fragments that further enhance cellular activation and inflammatory amplification [112, 113].

#### Interactions between synoviocytes and Th17/Treg cells

The functional imbalance between Th17 and Treg cells is a key driver of immune dysregulation in RA, wherein synoviocytes modulating their differentiation and function through cytokine secretion to establish a positive inflammatory feedback loop. IL-6 and IL-23 secreted by FLSs promote the differentiation of naïve T cells into Th17 cells while inhibiting Treg differentiation and function [114, 115]. Th17 cell-derived IL-17 A binds to interleukin-17 (IL-17) receptors on FLSs and activates STAT3- and NF-κB-associated signaling, thereby promoting further IL-6 and IL-23 secretion by FLSs. This reciprocally enhances Th17 cell proliferation and differentiation, thereby creating an FLS-Th17 positive feedback loop [116, 117]. Additionally, IL-17 A promotes MMP secretion by FLSs, thereby enhancing their invasive capacity and exacerbating cartilage and bone degradation [118, 119]. Although Treg cells can inhibit FLS activation through IL-10 and TGF-β secretion, IL-6 produced by FLSs in the RA synovial microenvironment suppresses Treg immunosuppressive function, impairing their ability to regulate inflammation and thereby exacerbating immune imbalance [120].

#### Interactions between synoviocytes and B cells

Interactions between synoviocytes and B cells, mediated primarily by autoantibodies and cytokines, contribute to inflammatory amplification and tissue destruction. Autoantibodies such as RF and anti-CCP antibodies secreted by B cells bind to antigens on synoviocytes to form immune complexes, which activate the complement system and generate C3a and C5a fragments. These fragments bind to complement receptors on type A synoviocytes and FLSs, activating pro-inflammatory programs and stimulating the secretion of pro-inflammatory cytokines and MMPs [121]. In turn, B cell-derived IL-6 and TNF-α activate FLSs and type A synoviocytes, driving bone and cartilage degradation, synovial hyperplasia, and angiogenesis [122]. Reciprocally, FLS-secreted BAFF promotes B cell proliferation and differentiation, enhancing antibody production and establishing a synoviocyte-B cell positive feedback loop [123]. Furthermore, B cells function as antigen-presenting cells that present synoviocyte-derived antigens to T cells, thereby activating T cell responses and further amplifying immune dysregulation.

### Direct interactions among non-synoviocyte populations: synergistic regulation of synovial microenvironmental homeostasis

**Fig. 4:**
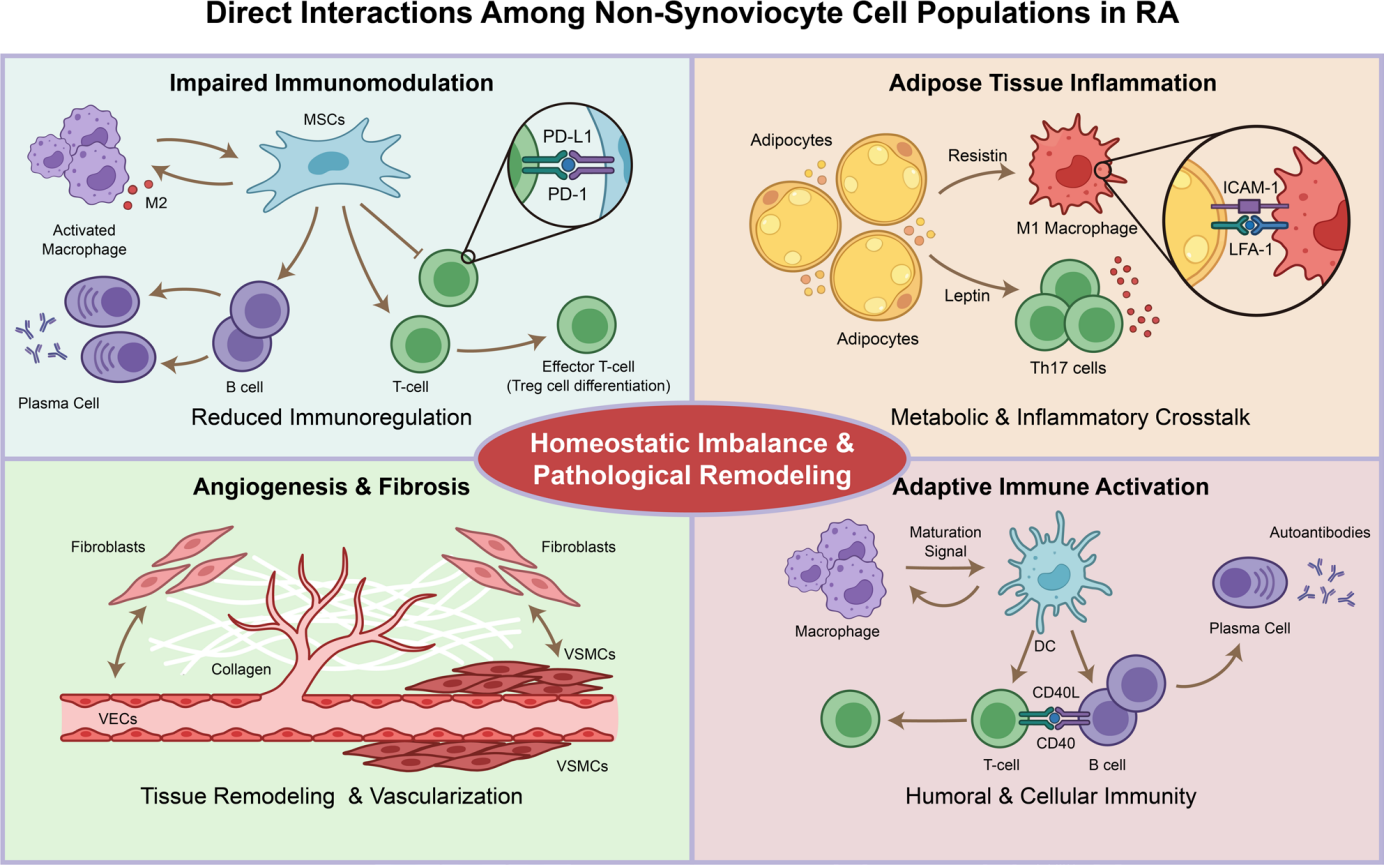
Direct interactions among non-synoviocyte populations in RA. This figure illustrates the direct interactions among non-synoviocyte populations in the RA synovial microenvironment and their synergistic roles in regulating inflammatory homeostasis. It depicts the immunomodulatory crosstalk between MSCs and immune cells, the pro-inflammatory communication between adipocytes and immune cells through adipokines and adhesion molecules, and the coupled angiogenic and fibrotic interactions among fibroblasts, VECs, and VSMCs. In parallel, direct interactions among macrophages, DCs, Th17 cells, Treg cells, and B cells contribute to persistent antigen presentation, immune imbalance, and autoantibody production. Together, these non-synoviocyte cell networks cooperate with synoviocyte-driven responses to amplify inflammation and tissue remodeling in RA. Abbreviations: RA, rheumatoid arthritis; MSCs, mesenchymal stem cells; VECs, vascular endothelial cells; VSMCs, vascular smooth muscle cells; DCs, dendritic cells; Th17, T helper 17 cells; Treg, regulatory T cells; IL, interleukin; TGF-β, transforming growth factor-β; IDO, indoleamine 2,3-dioxygenase; PD-L1, programmed death-ligand 1; VEGF, vascular endothelial growth factor; PDGF, platelet-derived growth factor

Beyond the crosstalk between synoviocytes and other cell types, direct interactions also occur among MSCs, fibroblasts, adipocytes, vascular-associated cells, and immune cells. These non-synoviocyte populations establish independent yet interconnected regulatory sub-networks through cytokine secretion, adhesion molecule expression, and signal transduction. These networks synergize with synoviocyte-mediated pathways to drive synovial microenvironment imbalance and promote RA progression. To integrate these non-synoviocyte interaction axes at the network level, Fig. 4 summarizes the direct crosstalk among mesenchymal, vascular-associated, adipose, and immune cell populations and illustrates how these coupled sub-networks cooperate with synoviocyte-driven responses to sustain inflammatory imbalance in RA.

#### Interactions between mesenchymal stem cells and immune cells: core regulation of immune homeostasis

MSCs interact with macrophages, T cells, and B cells within the synovial microenvironment through direct cell contact and paracrine signaling. Their core function involves modulating immune cell phenotype and function (e.g., inducing M2 macrophage polarization and Treg differentiation) through immune remodeling, thereby suppressing inflammatory cascades and maintaining local immune homeostasis [124–126]. However, in RA, MSC immunomodulatory capacity is impaired, limiting suppression of aberrant immune responses and potentially promoting a shift toward a pro-inflammatory phenotype. In interactions with macrophages, MSCs secrete molecules such as PGE2, hepatocyte growth factor (HGF), and IL-10 to inhibit polarization toward the pro-inflammatory M1 phenotype while promoting the anti-inflammatory M2 polarization [127, 128]. Conversely, IL-10 and TGF-β secreted by M2 macrophages enhance MSC immunosuppressive function, establishing a potential anti-inflammatory regulatory loop [126]. MSC-T cell interactions occur through direct contact and paracrine signaling. MSCs express IDO to degrade tryptophan, thereby depriving T cells of this essential amino acid and inhibiting aberrant proliferation [129]. Additionally, MSC-derived TGF-β and IL-10 promote differentiation of naïve T cells into Treg cells while suppressing Th17 differentiation and activation [130]. Moreover, programmed death-ligand 1 (PD-L1) on MSCs binds to programmed cell death protein 1 (PD-1) on T cells to deliver immunosuppressive signals, thereby attenuating T cell pro-inflammatory activity [125]. Regarding B cells, MSCs secrete c-x-c motif chemokine ligand 12 (CXCL12) to regulate migration and adhesion, and directly inhibit B cell proliferation and differentiation, thereby reducing plasma cell formation and autoantibody (e.g., RF, anti-CCP) secretion and mitigating immune complex-mediated inflammation [131–133]. However, in RA, B cell-derived IL-6 reciprocally impairs MSC immunomodulatory function, thereby disrupting this regulatory balance [134]. Overall, the MSC–M2 macrophage–Treg axis may constitute one of the principal cellular modules associated with synovial immune homeostasis and remission maintenance. Impairment of this axis in RA may weaken local immune restraint and thereby favor persistence of inflammatory activity.

#### Interactions between adipocytes and immune cells: the metabolic-inflammatory nexus

The interactions between adipocytes and immune cells represent a critical nexus linking metabolic dysregulation to immune inflammation and play a key role in sustained RA synovial inflammation [135]. Adipocytes in the sublining layer and adjacent fat pads modulate macrophage and T cell function through adipokine secretion or cell-cell contact, thereby establishing a complex inflammatory amplification network [136, 137]. A prototypical inflammatory positive feedback loop exists between adipocytes and macrophages. Adipocyte-derived pro-inflammatory adipokines such as leptin and resistin recruit peripheral monocytes to synovial tissue through chemotaxis and induce differentiation into pro-inflammatory M1 macrophages [138, 139]. Conversely, TNF-α and IL-1β secreted by M1 macrophages activate adipocytes to release additional pro-inflammatory mediators, thereby exacerbating local inflammation [140]. Additionally, inflammation-induced upregulation of ICAM-1 on adipocytes facilitates binding to LFA-1 on macrophages, enhancing contact-dependent intercellular signaling and amplifying inflammatory responses [141, 142]. Adipocyte-T cell interactions primarily affect T cell subset differentiation balance. Leptin activates the JAK-STAT3 pathway to promote differentiation of naïve T cells into Th17 cells while inhibiting Treg cell proliferation and function [143, 144]. Reciprocally, Th17 cell-derived IL-17 stimulates adipocytes to produce pro-inflammatory mediators, thereby creating an adipocyte-Th17 cell vicious cycle [145, 146]. Notably, the expression of anti-inflammatory adipokines (e.g., adiponectin) is markedly downregulated in RA tissues, leading to loss of inhibition of aberrant T cell activation and thereby exacerbating immune imbalance [147].

#### Interactions between fibroblasts and vascular-associated cells: coupled regulation of angiogenesis and fibrosis

Interactions among FLSs, VECs, and VSMCs constitute the central mechanism driving the coordinated angiogenesis-fibrosis process in RA synovium, collectively providing structural support and metabolic sustenance for synovial hyperplasia [148, 149]. Fibroblasts and VECs establish a synergistic regulatory loop: activated fibroblasts secrete abundant angiogenic factors, including VEGF, FGF-2, and PDGF, which directly stimulate VEC proliferation, migration, and lumen formation [150–152]. Additionally, fibroblast-derived MMPs degrade ECM, thereby facilitating neovessel extension [153, 154]. Reciprocally, VEC-secreted PDGF and TGF-β activate fibroblasts to synthesize matrix components, including collagen, thereby promoting synovial fibrosis [155, 156]. Furthermore, fibroblast surface integrin α5β1 binds to VEC-secreted fibronectin. This matrix-mediated interaction markedly enhances intercellular signal efficiency [157, 158]. The interactions between fibroblasts and VSMCs primarily drive vascular wall remodeling: fibroblast-derived TGF-β induces VSMCs to undergo phenotypic transition toward a myofibroblast-like state, thereby enhancing collagen synthesis, proliferation, and migration required for neovessel wall formation [159, 160]. In turn, VSMC-secreted Ang II activates the NF-κB pathway in fibroblasts, stimulating IL-6 and collagen secretion and establishing a fibroblast-VSMC pro-fibrotic loop that exacerbates pathological synovial remodeling [161, 162].

#### Interactions among immune cell subsets: sustaining the autoimmune response

Interactions among immune cell subsets are central to both the initiation and maintenance of the autoimmune response in RA. Interactions among macrophages and DCs, Th17 and Treg cells, and T and B cells establish positive feedback loops that breach immune tolerance and perpetuate inflammatory amplification [163, 164]. Macrophages and DCs synergistically regulate antigen presentation: macrophage-derived IL-1β and IL-6 promote the maturation of immature DCs by upregulating surface MHC class II and co-stimulatory molecules (CD80 and CD86) [165]. Conversely, mature DCs secrete pro-inflammatory cytokines and deliver antigen-presenting signals that promote T cell activation and subsequent macrophage M1 polarization, thereby amplifying the inflammatory cascade [166, 167]. The imbalance between Th17 and Treg cells is a hallmark of RA immune dysregulation: a cytokine-rich environment (e.g., elevated IL-6 and IL-23) promotes Th17 differentiation and IL-17 secretion while suppressing forkhead box protein P3 (FOXP3) expression and stability in Treg cells, potentially inducing their conversion into Th17-like phenotypes [168, 169]. This “Th17 expansion/Treg deficiency” imbalance prevents the synovial microenvironment from effectively suppressing inflammation through IL-10 and TGF-β, thereby sustaining chronic disease progression [170, 171]. Interactions between T cells and B cells represent a central mechanism underlying autoantibody production: CD40 ligand (CD40L) expressed on CD4⁺ T cells binds to CD40 on B cells, delivering critical co-stimulatory signals that drive B cell proliferation and differentiation into plasma cells secreting autoantibodies such as RF and anti-CCP [172, 173]. In turn, B cells function as efficient antigen-presenting cells that present processed self-antigens to T cells, creating a “T cell-B cell” reciprocal activation loop that sustains autoimmune responses [174, 175]. Therefore, these immune-cell interaction loops are relevant not only to the initiation and maintenance of autoimmunity, but also to the persistence of inflammatory activity in RA. Accordingly, inflammatory activity may be interpreted as a state in which pathogenic immune-cell circuits predominate over regulatory immune programs, thereby preventing effective restoration of synovial immune homeostasis.

### Dynamic transitions between inflammatory activity and remission

Beyond driving RA along the pathological sequence of early autoimmune initiation, middle-stage inflammatory amplification and synovial hyperplasia, and late fibrosis, pannus formation, and tissue destruction, cellular interactions within the synovial microenvironment also determine whether the joint remains in an inflammatory-active state, transiently rebalances, or enters a more durable remission-associated state. Inflammatory activity is characterized by the predominance of pathogenic cellular circuits centered on activated fibroblast-like synoviocytes (FLSs), M1-like macrophages, Th17 cells, B cells, and pro-inflammatory stromal responses, which collectively sustain cytokine amplification, immune dysregulation, synovial hyperplasia, and tissue injury. By contrast, remission should not be understood as the complete disappearance of inflammation, but rather as a relatively rebalanced microenvironment in which immunoregulatory programs mediated by M2-like macrophages, regulatory T (Treg) cells, and immunomodulatory mesenchymal stem cells (MSCs) regain functional dominance and restrain pathogenic circuits. Accordingly, remission failure reflects the inability of regulatory networks to durably overcome self-sustaining inflammatory loops, thereby allowing persistent inflammatory activity to evolve into progressive fibrosis, pannus stabilization, dysregulated bone remodeling, and irreversible structural damage.

## Key signaling pathways regulating synoviocyte interactions in RA

**Fig. 5:**
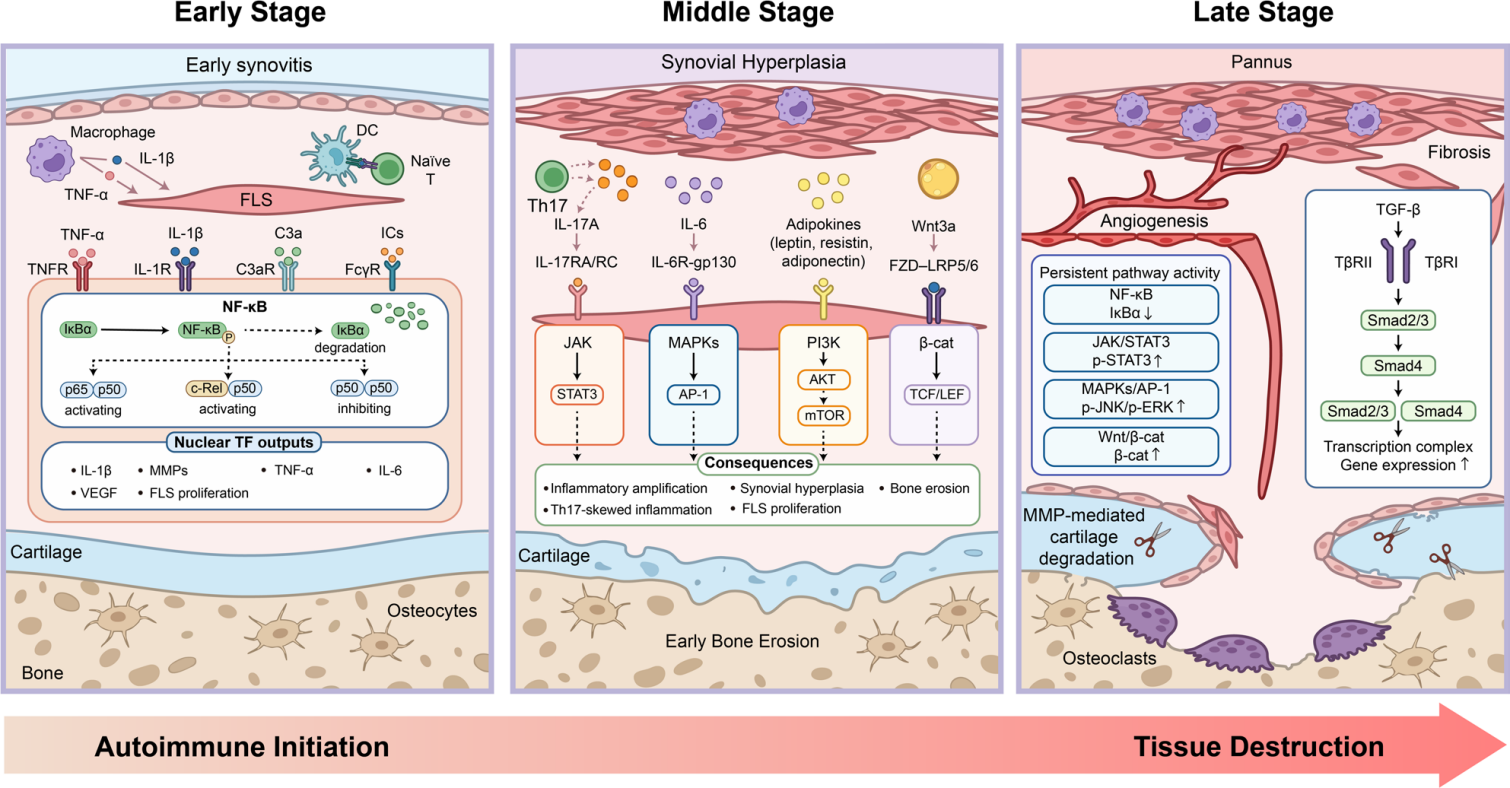
Stage-specific ligand–receptor–transcription factor programs driving synovial pathology during RA progression. This figure summarizes representative stage-dependent signaling events that translate extracellular ligand–receptor interactions into downstream transcription factor programs during RA progression. In the early stage, macrophage- and dendritic cell-associated inflammatory signals, including TNF-α/TNFR, IL-1β/IL-1R, C3a/C3aR, and immune complex/FcγR-related activation, stimulate canonical NF-κB signaling in FLSs. IκBα degradation permits the nuclear translocation of activating NF-κB dimers, including p65/p50 and c-Rel-containing complexes, whereas p50/p50 homodimers are shown as inhibitory transcriptional regulators. These NF-κB-dependent transcriptional outputs promote IL-1β, IL-6, TNF-α, VEGF, MMP expression, and FLS proliferation, thereby contributing to autoimmune initiation and early synovitis. In the middle stage, IL-17 A/IL-17RA/RC, IL-6/IL-6R-gp130, adipokine-associated signals, and Wnt3a-FZD/LRP5/6 signaling converge on JAK-STAT3, MAPK/AP-1, PI3K-AKT-mTOR, and β-catenin/TCF-LEF programs. These pathways sustain inflammatory amplification, Th17-skewed inflammation, FLS proliferation, synovial hyperplasia, and early bone erosion. In the late stage, persistent NF-κB, JAK/STAT3, MAPK/AP-1, and Wnt/β-catenin activity cooperates with TGF-β–TβRII/TβRI–Smad2/3/4 signaling to promote pannus formation, angiogenesis, MMP-mediated cartilage degradation, osteoclast activation, fibrosis, and irreversible tissue destruction. Together, this stage-oriented schematic illustrates how ligand–receptor signaling and downstream transcription factor programs coordinate the transition from autoimmune initiation to inflammatory amplification and late structural damage in RA. Abbreviations: RA, rheumatoid arthritis; FLSs, fibroblast-like synoviocytes; DCs, dendritic cells; TNF-α, tumor necrosis factor-alpha; TNFR, tumor necrosis factor receptor; IL-1β, interleukin-1 beta; IL-1R, interleukin-1 receptor; C3aR, C3a receptor; ICs, immune complexes; FcγR, Fc gamma receptor; NF-κB, nuclear factor kappa-light-chain-enhancer of activated B cells; IκBα, inhibitor of nuclear factor kappa-B alpha; c-Rel, REL proto-oncogene; VEGF, vascular endothelial growth factor; MMPs, matrix metalloproteinases; IL-6R, interleukin-6 receptor; gp130, glycoprotein 130; IL-17RA/RC, interleukin-17 receptor A/C; FZD, Frizzled receptor; LRP5/6, low-density lipoprotein receptor-related protein 5/6; JAK-STAT, Janus kinase/signal transducer and activator of transcription; STAT3, signal transducer and activator of transcription 3; MAPK, mitogen-activated protein kinase; AP-1, activator protein-1; PI3K, phosphoinositide 3-kinase; AKT, protein kinase B; mTOR, mechanistic target of rapamycin; β-cat, β-catenin; TCF/LEF, T-cell factor/lymphoid enhancer-binding factor; TGF-β, transforming growth factor-beta; TβRI, transforming growth factor-beta type I receptor; TβRII, transforming growth factor-beta type II receptor; Smad, mothers against decapentaplegic homolog

Intercellular interactions within the RA synovial microenvironment are translated into stage-specific pathological outcomes through interconnected signaling pathways that regulate cellular activation, proliferation, differentiation, migration, and cytokine production. These pathways do not merely mediate isolated molecular events; rather, they function as mechanistic switches that determine whether the synovium undergoes early autoimmune initiation, progresses into middle-stage inflammatory amplification and synovial hyperplasia, or advances toward late fibrosis, pannus formation, and irreversible tissue destruction. Moreover, by shaping the balance between pathogenic and immunoregulatory cellular programs, these signaling networks also influence whether the local microenvironment remains in an inflammatory-active state, transiently rebalances, or fails to achieve durable remission. Accordingly, pathways such as NF-κB, MAPK, JAK-STAT, and TGF-β/Smad should be viewed as integrated regulators of both pathological stage progression and clinical state transitions in RA. To connect cellular interaction patterns with their intracellular mechanistic basis, Fig. 5 summarizes the dominant signaling programs that emerge across different stages of RA progression. Rather than representing a static collection of pathways, this stage-oriented schematic highlights how inflammatory, proliferative, and fibrotic signaling programs become differentially dominant as the synovial microenvironment shifts from autoimmune initiation to chronic hyperplasia and finally to tissue-destructive remodeling.

With this foundation, the following sections examine the major signaling pathways in greater detail, beginning with NF-κB as a central mediator of early inflammatory activation and sustained synoviocyte pathogenicity.

### Core inflammatory pathway: the NF-κB pathway

The NF-κB pathway is a core inflammatory hub in the RA synovial microenvironment, extensively participating in signal transduction and cellular activation across diverse synoviocyte interactions and thereby linking early autoimmune initiation to middle-stage inflammatory amplification and, ultimately, to late pannus-supported tissue injury. The NF-κB family comprises subunits including the RELA proto-oncogene (p65), the RELB proto-oncogene, the REL proto-oncogene, the nuclear factor kappa B subunit 1 (p50, processed from its precursor p105), and the nuclear factor kappa B subunit 2 (p52, processed from its precursor p100). Under basal conditions, these subunits are sequestered in the cytoplasm in an inactive state through association with the inhibitory protein inhibitor of kappa B (IκB) [176]. Upon external stimulation (e.g., by TNF-α, IL-1β, C3a, or immune complexes), the IκB kinase (IKK) complex phosphorylates IκB, triggering its ubiquitin-mediated degradation and subsequent release of NF-κB subunits, thereby initiating inflammatory signaling in synoviocytes. The activated subunits then translocate to the nucleus, where they regulate transcription of IL-6, TNF-α, MMP-3, VEGF, and other pathogenic mediators, thereby converting early inflammatory stimulation into a broader synovial program of cytokine amplification, stromal activation, angiogenesis, and progressive tissue injury [177]. Beyond this general activation process, the transcriptional output of NF-κB signaling may also be shaped by the specific composition of NF-κB dimers. In canonical inflammatory signaling, p65/p50 heterodimers are commonly involved in the transcriptional activation of pro-inflammatory mediators, whereas c-Rel-containing dimers appear to participate more selectively in immune-cell activation, cytokine transcription, and T-cell polarization. Recent evidence from TLR7-driven inflammatory models indicates that c-Rel deficiency can alter the formation of activating NF-κB dimers and favor inhibitory p50 homodimer binding at the IL-1β and IL-6 promoters, thereby reducing inflammatory cytokine expression and Th17-polarizing capacity [178]. Although these findings were mainly derived from psoriasis-related dendritic-cell inflammation, they provide a useful mechanistic reference for understanding how NF-κB dimer composition may regulate inflammatory intensity within autoimmune microenvironments. Consistently, c-Rel deficiency has also been associated with reduced susceptibility to collagen-induced arthritis, suggesting that distinct NF-κB subunits may play non-redundant roles in autoimmune initiation, synovial inflammatory amplification, and local joint destruction [179]. From this perspective, NF-κB signaling in RA should not be regarded only as a globally activated inflammatory pathway, but also as a transcription factor network whose subunit composition may influence whether synovial inflammation remains self-sustaining or becomes more amenable to therapeutic modulation.

NF-κB activation is pervasive in synoviocyte interactions and contributes to multiple stages of RA progression, making it one of the principal pathways that links early autoimmune initiation to middle-stage inflammatory amplification and, ultimately, to late pannus-associated structural damage, through the following mechanisms: (1) during the early autoimmune initiation and subsequent inflammation amplification phases, in type A synoviocyte-FLS interactions, FLS-secreted C3a binds to C3aR on type A synoviocytes, activating NF-κB and promoting IFN-β secretion [66]; simultaneously, TNF-α and IL-1β derived from type A synoviocytes activate NF-κB in FLSs, stimulating pro-inflammatory cytokine and MMP production and thereby reinforcing inflammatory amplification [180]; (2) In immune-synoviocyte interactions that sustain the middle stage of disease, IL-17 A from Th17 cells and TNF-α from macrophages activate NF-κB in FLSs, thereby enhancing their pro-inflammatory activity [181, 182]. Additionally, B cell-derived immune complexes activate this pathway in synoviocytes through complement receptors, further amplifying inflammatory signaling [183]; (3) in synoviocyte-vascular cell interactions during the late pannus formation stage, FLS-secreted VEGF activates NF-κB in VECs, promoting endothelial proliferation and angiogenesis and thereby supporting pannus expansion [184]. Furthermore, NF-κB activation suppresses the multilineage differentiation potential of MSCs while enhancing their pro-inflammatory activity, thereby exacerbating synovial inflammation and synovial hyperplasia [185]. Substantial evidence indicates that aberrant NF-κB activation is a key molecular basis for persistent inflammatory activity in RA, since it stabilizes pro-inflammatory cellular crosstalk, limits restoration of local immune restraint, and thereby increases the likelihood of remission failure and subsequent structural progression. Inhibitors targeting key molecules such as IKK and p65 markedly suppress synoviocyte activation and cytokine secretion, thereby attenuating joint inflammation and tissue destruction in animal models and supporting the idea that interruption of this pathway may help restore control over inflammatory activity before irreversible structural damage becomes established. These findings highlight the NF-κB pathway as a critical therapeutic target for controlling inflammatory activity in RA [186, 187].

### Regulation of proliferation and migration: the MAPK pathway

The MAPK pathway, which comprises the extracellular signal-regulated kinase (ERK), p38 MAPK, and c-Jun N-terminal kinase (JNK) subfamilies, is a critical regulator of cell proliferation, migration, differentiation, and cytokine secretion, and it plays a pivotal role in converting middle-stage inflammatory amplification into synovial hyperplasia, invasive behavior, and subsequent structural damage in RA [188]. Upon stimulation by cytokines, growth factors, or ECM components, membrane receptors activate upstream kinases (e.g., rapidly accelerated fibrosarcoma [Raf] and mitogen-activated protein kinase kinase [MEK]), which subsequently phosphorylate ERK, p38, or JNK and initiate downstream signaling cascades. These activated MAPKs translocate to the nucleus, where they phosphorylate transcription factors (e.g., activator protein-1 [AP-1] and E26 transformation-specific like-1 protein [Elk-1]) and regulate gene transcription associated with synoviocyte proliferation and migration. In synoviocyte interactions, MAPK signaling primarily regulates proliferation and migration, thereby acting as a key mechanistic bridge between middle-stage synovial hyperplasia and late structural injury through the following mechanisms: (1) during the late pannus formation stage, FLS-secreted FGF-2 binds to fibroblast growth factor receptors (FGFRs) on VECs, activating the ERK-MAPK pathway and driving VEC proliferation, migration, and lumen formation to promote angiogenesis. Reciprocally, VEC-derived PDGF activates the ERK-MAPK pathway in FLSs, thereby stimulating their proliferation and contributing to synovial hyperplasia in the middle stage [188]; (2) in synoviocyte-fibroblast interactions, FLS-secreted TGF-β activates the p38-MAPK pathway in fibroblasts, promoting proliferation and collagen synthesis and thereby exacerbating synovial fibrosis during the late remodeling stage [122]; (3) in immune-synoviocyte interactions, macrophage-derived TNF-α activates the p38-MAPK and JNK pathways in FLSs, stimulating MMP-9 secretion and enhancing matrix degradation characteristic of the late tissue destruction stage [189]. Similarly, Th17 cell-derived IL-17 A activates the JNK pathway in FLSs, promoting proliferation and invasion and further accelerating synovial expansion and tissue damage [122]. Furthermore, substantial crosstalk exists between the MAPK and NF-κB pathways, and this cooperation is particularly important because it stabilizes self-sustaining inflammatory circuits within the synovial microenvironment, thereby making active disease states more difficult to extinguish. For example, p38 MAPK can phosphorylate the IKK complex, promoting NF-κB nuclear translocation and pro-inflammatory gene transcription, thereby further amplifying inflammatory responses within the synovium [190]. Collectively, MAPK signaling integrates inflammatory stimulation with synoviocyte proliferation, migration, and matrix degradation, thereby linking middle-stage synovial hyperplasia to late structural destruction while also helping explain why pathogenic stromal activation remains difficult to reverse once the synovial microenvironment has entered a self-sustaining inflammatory state.

### Cytokine-mediated signaling pathway: the JAK-STAT pathway

The JAK-STAT pathway is a central mediator of cytokine signaling that extensively regulates interactions between RA synoviocytes and immune cells, thereby driving immune imbalance and inflammatory amplification, particularly during the inflammatory active state and middle stage of disease, when remission-associated regulatory programs are progressively lost [191]. The JAK family comprises Janus kinase 1 (JAK1), JAK2, Janus kinase 3 (JAK3), and tyrosine kinase 2 (TYK2), whereas the STAT family includes signal transducer and activator of transcription 1 (STAT1), signal transducer and activator of transcription 2 (STAT2), STAT3, signal transducer and activator of transcription 4 (STAT4), signal transducer and activator of transcription 5 (STAT5; subtypes 5a and 5b), and signal transducer and activator of transcription 6 (STAT6) [192, 193]. Upon cytokine binding, receptors dimerize to activate associated intracellular JAK kinases, which phosphorylate tyrosine residues within the receptor intracellular domains, thereby creating STAT-binding sites and initiating downstream cytokine-dependent signaling. Recruited STAT molecules are subsequently phosphorylated by JAKs, dimerize, and translocate to the nucleus to regulate gene transcription associated with inflammatory activation, immune-cell differentiation, and synoviocyte pathogenic behavior, thereby directly shaping whether the local microenvironment remains dominated by pathogenic immune programs or regains partial immunoregulatory restraint [194]. In RA, the JAK-STAT pathway plays a fundamental role in regulating immune cell differentiation and synoviocyte activation during the middle inflammation amplification phase, thereby acting as a key molecular driver of cytokine-mediated inflammatory persistence and remission failure through the following mechanisms: (1) in FLS-Th17/Treg interactions, FLS-derived IL-6 binds to IL-6 receptors on naïve CD4⁺ T cells to activate the JAK1/STAT3 pathway, promoting naïve T cell differentiation into Th17 cells while suppressing Treg differentiation and thereby reinforcing immune imbalance [195]; reciprocally, Th17-derived IL-17 A can also interact with JAK-STAT-related signaling in FLSs in certain contexts, while its better-established downstream effects involve NF-κB and MAPK activation, enhancing proliferation and MMP secretion and thereby establishing a positive feedback loop that sustains the inflammatory amplification phase [193]; (2) in synoviocyte-adipocyte interactions, adipocyte-derived leptin activates the JAK2/STAT3 pathway in FLSs, thereby augmenting their pro-inflammatory activity and contributing to cytokine-driven synovial activation; (3) in immune-synoviocyte interactions, macrophage-derived IL-6 activates STAT3 to promote inflammatory responses, whereas IL-10 activates STAT3/STAT5 to exert anti-inflammatory effects; however, within the RA synovial microenvironment, pro-inflammatory signaling predominates and thereby sustains chronic immune dysregulation and inflammatory activity [48]. Aberrant JAK-STAT activation represents a critical mechanism underlying RA immune dysregulation and is closely associated with the maintenance of inflammatory activity, since it reinforces Th17-dominant and pro-inflammatory stromal programs while weakening the cellular conditions required for durable remission. Clinically approved JAK inhibitors (e.g., tofacitinib and baricitinib) effectively alleviate joint inflammation by blocking cytokine-mediated signaling, thereby validating this pathway as a therapeutic target not only for controlling inflammatory activity but also for rebalancing pathogenic versus regulatory immune programs in favor of more durable disease control [196–198].

### Regulation of fibrosis and differentiation: the TGF-β/Smad pathway

The TGF-β/Smad pathway is a pivotal regulator of cell proliferation, differentiation, ECM synthesis, and fibrosis, playing a central role in RA synovial fibrosis, cellular differentiation imbalance, and the transition from inflammatory remodeling to fibrotic and structurally destructive disease; importantly, its dominance also indicates that the synovial microenvironment is shifting from a relatively plastic inflammatory state toward a less reversible fibrotic and structurally destructive state [199]. The TGF-β family comprises three isoforms TGF-β1, TGF-β2, and TGF-β3. Signaling is initiated when TGF-β binds to the type II receptor (TβRII), which recruits and phosphorylates the type I receptor (TβRI). Activated TβRI subsequently phosphorylates mothers against decapentaplegic homolog 2 and 3 (Smad2/3), which form complexes with mothers against decapentaplegic homolog 4 (Smad4) and translocate to the nucleus to regulate target genes associated with fibrosis, differentiation, and stromal remodeling, thereby shifting the synovial microenvironment toward a less reversible fibrotic and structurally destructive state. Mothers against decapentaplegic homolog 6 (Smad6) and mothers against decapentaplegic homolog 7 (Smad7) function as inhibitory regulators that suppress TGF-β/Smad pathway activation [200, 201]. In RA, the TGF-β/Smad pathway primarily regulates fibrosis and cellular differentiation, acting as a core driver during the late stage of pannus formation, fibrotic remodeling, dysregulated bone remodeling, and irreversible structural destruction through the following mechanisms: (1) FLS-secreted TGF-β1 activates Smad2/3 in fibroblasts, promoting proliferation and collagen (types I and III) synthesis and thereby exacerbating synovial fibrosis and irreversible joint remodeling; activated fibroblasts subsequently secrete additional TGF-β1, establishing a positive feedback loop [202, 203]; (2) in synoviocyte-MSC interactions, FLS-secreted TGF-β1 inhibits MSC differentiation into osteoblasts and adipocytes through Smad-dependent signaling, thereby maintaining MSCs in a proliferative, pro-inflammatory state and impairing restoration of tissue homeostasis [204]; (3) in synoviocyte-vascular interactions, TGF-β1 regulates VSMC proliferation and differentiation through the Smad pathway, thereby driving neovessel wall remodeling to support pannus expansion [205, 206]. Moreover, substantial crosstalk exists between the TGF-β/Smad and MAPK pathways, further reinforcing fibroproliferative remodeling in advanced RA and helping drive the transition from late inflammatory remodeling to fixed pannus-associated structural damage. For example, TGF-β1 can activate ERK-MAPK signaling to synergistically promote synovial fibroblast proliferation and fibrosis [207]. Within the RA synovial microenvironment, TGF-β/Smad activation closely correlates with fibrosis severity and late-stage structural remodeling, indicating that this pathway is a key molecular marker of declining tissue plasticity and progression toward irreversible joint damage. Inhibitors targeting TβRI or Smad2/3 significantly attenuate synovial fibrosis, highlighting a potential therapeutic avenue for limiting late-stage tissue remodeling and reducing the accumulation of irreversible structural injury once inflammatory disease has entered a fibrotic remodeling phase [208]. These findings further suggest that late-stage structural progression in RA reflects not only persistent pannus-associated inflammation and fibrotic remodeling, but also progressive failure of bone remodeling, characterized by impaired osteoblast-mediated repair and enhanced osteoclast-driven bone resorption.

### Additional key signaling pathways and pathway crosstalk

**Fig. 6:**
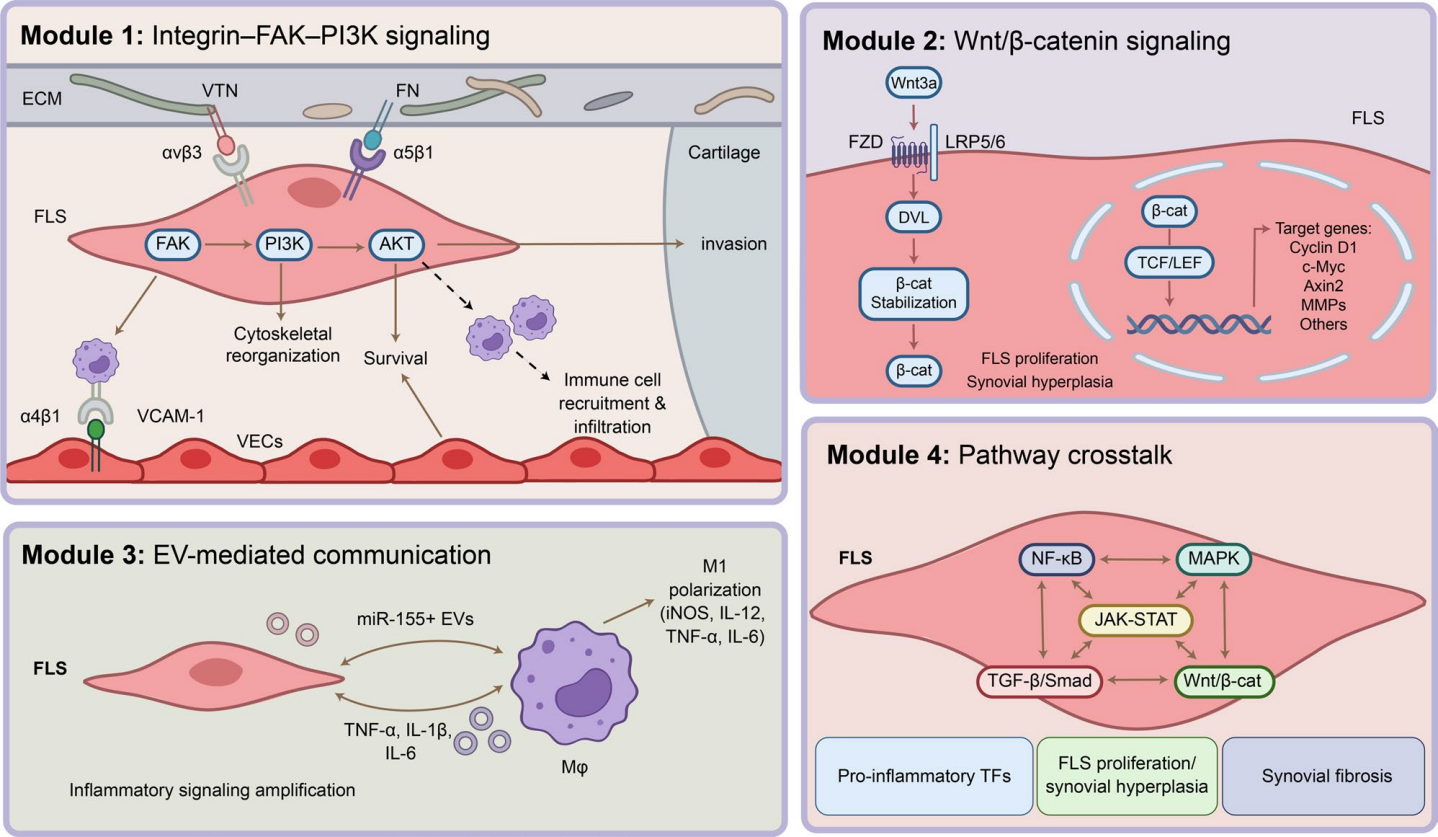
Additional signaling pathways, selected ligand–receptor axes, and pathway crosstalk in the RA synovial microenvironment. This figure illustrates additional signaling modules and pathway crosstalk that reinforce pathogenic cellular communication in the RA synovial microenvironment. Module 1 shows integrin–FAK–PI3K signaling in FLSs. ECM components, including vitronectin and fibronectin, engage integrins such as αvβ3 and α5β1, respectively, leading to FAK activation and downstream PI3K-AKT signaling. This pathway promotes cytoskeletal reorganization, FLS survival, and cartilage invasion. The VCAM-1/α4β1 (VLA-4) adhesion axis between VECs and immune cells is also shown to indicate how adhesion-dependent interactions contribute to immune-cell recruitment and infiltration. Module 2 depicts Wnt/β-catenin signaling. Wnt3a binding to the FZD/LRP5/6 receptor complex activates DVL, stabilizes β-catenin, and promotes β-catenin nuclear translocation. Nuclear β-catenin cooperates with TCF/LEF transcription factors to induce target genes, including Cyclin D1, c-Myc, Axin2, and MMPs, thereby supporting FLS proliferation, synovial hyperplasia, and matrix remodeling. Module 3 illustrates EV-mediated communication between FLSs and macrophages. FLS-derived miR-155-enriched EVs promote macrophage M1 polarization, characterized by increased iNOS, TNF-α, and IL-6, whereas macrophage-derived inflammatory mediators, including TNF-α, IL-1β, and IL-6, reinforce FLS activation and inflammatory signaling amplification. Module 4 summarizes pathway crosstalk among NF-κB, MAPK, JAK-STAT, TGF-β/Smad, and Wnt/β-catenin signaling. These interconnected pathways cooperatively enhance pro-inflammatory transcription factor activity, FLS proliferation, synovial hyperplasia, and synovial fibrosis. Collectively, this figure emphasizes that RA synovial pathology is driven not only by individual signaling cascades, but also by matched ligand–receptor systems, adhesion-dependent interactions, EV-mediated cargo transfer, and convergent pathway crosstalk. Abbreviations: RA, rheumatoid arthritis; FLSs, fibroblast-like synoviocytes; ECM, extracellular matrix; FN, fibronectin; VTN, vitronectin; FAK, focal adhesion kinase; PI3K, phosphoinositide 3-kinase; AKT, protein kinase B; VECs, vascular endothelial cells; VCAM-1, vascular cell adhesion molecule 1; α4β1, very late antigen-4; FZD, Frizzled receptor; LRP5/6, low-density lipoprotein receptor-related protein 5/6; DVL, Dishevelled; β-cat, β-catenin; TCF/LEF, T-cell factor/lymphoid enhancer-binding factor; EVs, extracellular vesicles; miR-155, microRNA-155; Mφ, macrophage; iNOS, inducible nitric oxide synthase; TNF-α, tumor necrosis factor-alpha; IL-1β, interleukin-1 beta; IL-6, interleukin-6; NF-κB, nuclear factor kappa-light-chain-enhancer of activated B cells; MAPK, mitogen-activated protein kinase; JAK-STAT, Janus kinase/signal transducer and activator of transcription; TGF-β, transforming growth factor-beta; Smad, mothers against decapentaplegic homolog; TFs, transcription factors

Extending these mechanistic programs, Fig. 6 further integrates additional signaling modules and pathway crosstalk in the RA synovial microenvironment, highlighting integrin-FAK-PI3K signaling, Wnt/β-catenin activity, extracellular vesicle-mediated intercellular communication, and the cooperative interactions among NF-κB, MAPK, JAK-STAT, and TGF-β-related pathways. Beyond the core pathways discussed above, RA synoviocyte interactions also involve additional signaling cascades that function in a stage-dependent manner to connect early inflammatory activation with middle-stage synovial expansion and, ultimately, late pannus-associated tissue destruction. Among them, the Integrin-FAK-PI3K pathway is particularly important for cell adhesion, migration, and invasion during the transition from persistent synovial hyperplasia to invasive tissue destruction. Integrins expressed on FLSs, such as α5β1 and αvβ3, bind to ECM components, including fibronectin and vitronectin, thereby activating FAK and promoting adhesion-dependent signaling. Activated FAK subsequently activates the PI3K-AKT pathway to drive FLS migration and invasion into cartilage, thereby promoting the conversion of chronic stromal activation into irreversible late-stage structural damage [209]. Concurrently, VLA-4 (α4β1)-expressing activated immune cells interact with VCAM-1-rich synovial/endothelial compartments, facilitating cell recruitment and retention within the inflamed joint, strengthening intercellular adhesion through FAK-PI3K signaling and thereby facilitating immune cell infiltration, stabilizing inflammatory activity, and reducing the likelihood that the synovial microenvironment will revert to a remission-associated state [148]. The Wnt/β-catenin pathway regulates FLS proliferation and is particularly associated with the middle amplification stage, during which it helps convert inflammatory stimulation into sustained synovial hyperplasia and pathogenic stromal expansion. Aberrant activation promotes synovial hyperplasia during the middle amplification phase, and elevated levels of Wnt-related molecules such as Wnt family member 3 A (Wnt3a) and β-catenin in RA tissues suggest that inhibition of this pathway may markedly suppress FLS proliferation, preserve tissue plasticity during the middle stage, and potentially delay progression toward structurally destructive disease [210]. Mechanistically, Wnt3a binding to FZD/LRP5/6 receptors stabilizes cytoplasmic β-catenin and promotes its nuclear translocation. Nuclear β-catenin cooperates with T-cell factor/lymphoid enhancer-binding factor (TCF/LEF) transcription factors to induce genes associated with cell-cycle progression, stromal expansion, and invasive behavior. Therefore, Wnt3a/β-catenin signaling may represent not only a proliferative pathway but also a transcriptional program that preserves pathogenic FLS activation during the therapeutically plastic middle stage of RA. Signaling mediated by extracellular vesicles (including exosomes and microvesicles) has emerged as an important mode of intercellular communication in RA and may participate across multiple stages of disease progression by dynamically transmitting pathogenic cues between stromal, immune, and vascular cells. FLSs, macrophages, and vascular cells secrete exosomes containing bioactive cargos (e.g., microRNAs [miRNAs], messenger RNAs [mRNAs], and proteins) that regulate target cell function and reinforce intercellular pathogenic signaling, thereby helping maintain inflammatory activity and propagate maladaptive cellular programs across the synovial microenvironment [211]. FLS-derived exosomes enriched in miR-155 promote macrophage polarization toward the M1 phenotype, thereby shifting the local cellular balance away from immune regulation and toward persistent inflammatory activity [212]. Reciprocally, macrophage-derived exosomes transport TNF-α to FLSs, activating the NF-κB pathway and enhancing pro-inflammatory activity, thereby reinforcing a bidirectional macrophage-FLS circuit that favors remission failure and progressive synovial damage [213].

Importantly, these signaling pathways do not function in isolation but instead form an integrated regulatory network through extensive crosstalk that drives RA pathogenesis in a stage-dependent manner and helps determine whether the synovial microenvironment remains persistently inflamed, partially rebalances, or progresses toward irreversible structural remodeling. For example, the NF-κB and MAPK pathways reciprocally activate through IKK-p38 interactions, synergistically enhancing pro-inflammatory gene transcription and reinforcing inflammatory amplification, thereby helping stabilize the active disease state once self-sustaining inflammatory loops are established [214]. The JAK-STAT and NF-κB pathways co-regulate FLS proliferation and cytokine secretion through STAT3-p65 interactions, thereby contributing not only to sustained inflammatory activity but also to the persistence of pathogenic stromal programs that undermine durable remission [215]. Additionally, the TGF-β/Smad and Wnt/β-catenin pathways synergistically promote synovial fibrosis through Smad4-β-catenin complex formation, thereby accelerating the shift from late inflammatory remodeling to fixed fibrotic change, pannus stabilization, and irreversible structural injury [216, 217]. The complexity of this pathway crosstalk poses challenges for understanding RA pathogenesis; however, it also highlights an important translational principle: since early and middle disease stages may still retain partial cellular plasticity, stage-adapted and multi-target interventions aimed at these interacting pathways may promote remission, delay disease progression, and reduce irreversible tissue damage.

## Research advances and future perspectives

Recent advances in high-resolution and spatially resolved technologies have greatly refined our current understanding of the RA synovial microenvironment. Single-cell RNA sequencing has revealed substantial heterogeneity among fibroblast-like synoviocytes, macrophages, lymphocytes, and other stromal cell populations [218, 219], while spatial transcriptomics and imaging-based approaches have begun to define how these subsets are distributed and functionally organized within inflamed synovial tissue [220, 221]. Collectively, these developments have shifted the field from descriptive characterization of individual cell populations toward a more integrated view of how cellular states, spatial context, and intercellular communication jointly regulate inflammatory activity, remission, and tissue remodeling in RA.

The evidence discussed in this review supports the concept that RA progression is driven not by isolated pathogenic cell types but by a dynamic, stage-dependent interaction network involving stromal, immune, vascular, and metabolic components. Within this framework, early autoimmune initiation, the middle stage of inflammatory amplification and synovial hyperplasia, and late fibrosis, pannus formation, and structural destruction may be regarded as interconnected phases within a continuous pathological process. This view also provides a plausible explanation for clinical heterogeneity. Persistent inflammatory activity, incomplete disease control, remission instability, and progressive structural damage may all represent distinct manifestations of an imbalanced synovial microenvironment in which pathogenic cellular circuits continue to outweigh immunoregulatory programs. By contrast, remission is better understood not as the complete absence of inflammatory signals, but as a relatively rebalanced state in which regulatory networks regain sufficient capacity to restrain self-sustaining pathogenic loops.

**Table 1:** Key cellular interaction axes, molecular mediators, and targeted intervention strategies in the RA synovial microenvironment

Interaction Axis	Interacting Cells	Key Mediators	Signaling Pathways	Interventions (Current/Potential)	References
Resident Synovial Cell Interaction	Type A synoviocytes/STMs ↔ FLSs	TNF-α; IL-1β; IL-6; C3a; GM-CSF; ICAM-1;	NF-κB; JAK-STAT; C3aR signaling;	Current: TNF inhibitors (e.g., adalimumab, etanercept, infliximab); IL-6R antagonists (e.g., tocilizumab, sarilumab); Potential/Investigational: C3aR antagonists; GM-CSF inhibitors;	Mizoguchi F, et al. [76]
Synoviocyte-Immune Cell Interaction	FLSs ↔ Th17/Treg/B cells	IL-17 A; IL-23; IL-6; BAFF; CD40L; Autoantibodies;	JAK-STAT; NF-κB; PI3K-AKT signaling;	Current: JAK inhibitors (e.g., tofacitinib, baricitinib, upadacitinib); Anti-CD20 therapy (e.g., rituximab); Potential/Investigational: IL-17 pathway blockade (evidence remains mixed and these agents are not approved for RA); STAT3 inhibitors;	Tu J, et al. [222] Komatsu N, et al. [223]
Synoviocyte-Adipocyte Interaction	FLSs ↔ Adipocytes	Leptin; Resistin; Chemerin; Adiponectin;	JAK2-STAT3; NF-κB; MAPK;	Potential/Investigational: Leptin receptor antagonists; Metabolic regulators; Anti-chemerin agents;	Zhao CW, et al. [90] González-Rodríguez M, et al. [93]
Synoviocyte-Vascular Cell Interaction	FLSs ↔ VECs/VSMCs	VEGF; FGF-2; PDGF; Ang II; Tie2;	MAPK; NF-κB; Notch signaling;	Potential/Investigational: anti-angiogenic strategies targeting VEGF-related signaling; Multi-target kinase inhibitors;	Elshabrawy HA, et al. [95] Zhao F, et al. [224]
Synoviocyte-Fibroblast Interaction	FLSs ↔ Sublining Fibroblasts	TGF-β1; Cadherin-11; Fibronectin; Collagen;	TGF-β/Smad; Wnt/β-catenin; p38 MAPK;	Potential/Investigational: Anti-TGF-β agents; Wnt pathway inhibitors; Anti-cadherin-11 monoclonal antibodies;	Croft AP, et al. [61]
Synoviocyte-MSC Interaction	Synoviocytes ↔ MSCs	IL-1β; TNF-α (inhibitory); IL-10; Indoleamine 2,3-dioxygenase (IDO) (protective);	NF-κB; TGF-β/Smad;	Under Clinical Investigation: MSC Transplantation; Potential/Investigational: gene-modified or preconditioned MSCs;	Gao YF, et al. [225]

This table summarizes the six principal cellular interaction axes within the RA synovial microenvironment, including interactions between resident synoviocytes, synoviocytes and immune cells, synoviocytes and adipocytes, synoviocytes and vascular cells, and synoviocytes and fibroblasts/stem cells. It outlines the major molecular mediators and representative signaling pathways involved in each interaction axis, such as TNF-α, IL-6, VEGF, leptin, Cadherin-11, NF-κB, JAK-STAT, and MAPK. In addition, the table lists therapeutic approaches that are either currently used in RA management or are being explored as potential intervention strategies in preclinical studies, clinical trials, or translational research. These entries are intended to illustrate the therapeutic relevance of pathogenic cellular crosstalk, rather than to imply that all listed agents or targets have been approved or clinically validated for RA

Abbreviation: AKT, protein kinase B; BAFF, B-cell activating factor; FGF-2, fibroblast growth factor 2; FLSs, fibroblast-like synoviocytes; GM-CSF, granulocyte-macrophage colony-stimulating factor; IL-1β, interleukin-1 beta; IL-6, interleukin-6; IL-10, interleukin-10; IL-17A, interleukin-17A; IL-23, interleukin-23; JAK-STAT, Janus kinase/signal transducer and activator of transcription; MAPK, mitogen-activated protein kinase; MSCs, mesenchymal stem cells; NF-κB, nuclear factor kappa-light-chain-enhancer of activated B cells; PDGF, platelet-derived growth factor; PI3K, phosphoinositide 3-kinase; RA, rheumatoid arthritis; Smad, mothers against decapentaplegic homolog; STAT3, signal transducer and activator of transcription 3; STMs, synovial tissue macrophages; TGF-β, transforming growth factor-beta; Th17, T helper 17; TNF-α, tumor necrosis factor-alpha; Treg, regulatory T cells; VECs, vascular endothelial cells; VEGF, vascular endothelial growth factor; VSMCs, vascular smooth muscle cells.

An important implication of this framework is that the synovial microenvironment can be translated into a more actionable therapeutic model through the principal cellular interaction axes summarized in Table 1. These include six principal interaction axes: resident synoviocyte interaction, synoviocyte-immune cell interaction, synoviocyte-adipocyte interaction, synoviocyte-vascular cell interaction, synoviocyte-fibroblast interaction, and synoviocyte-MSC interaction. Rather than representing purely descriptive categories, these axes define partially overlapping pathogenic modules associated with shared mediators and signaling pathways, including TNF-α, IL-6, VEGF, leptin, Cadherin-11, NF-κB, JAK-STAT, and MAPK. Accordingly, Table 1 provides a useful bridge between synovial cellular crosstalk and potential intervention strategies, and underscoring that effective treatment may require disruption of pathogenic interaction networks rather than inhibition of single molecules in isolation.

This network-based perspective may also help explain why durable remission remains difficult to achieve in a substantial proportion of patients despite the availability of multiple targeted therapies. Once stromal activation, immune amplification, vascular remodeling, and metabolic-inflammatory signaling become mutually reinforcing, suppression of a single mediator or pathway may be insufficient to dismantle the broader pathogenic network. Residual crosstalk may continue to sustain inflammatory activity, support synovial hyperplasia, and promote progression toward structural injury even when one dominant pathway has been partially controlled. Moreover, although major signaling hubs such as NF-κB, MAPK, JAK-STAT, and TGF-β/Smad have been extensively characterized, the critical nodes governing pathway crosstalk, network resilience, and transitions between inflammatory activity, remission-associated control, and irreversible remodeling remain incompletely defined.

A further implication is that therapeutic priorities may need to be adjusted according to disease stage. In the early stage, when autoimmune activation is being established but key cellular subsets and interaction programs may still retain substantial plasticity, treatment goals should extend beyond suppression of overt inflammation to include restoration of immune homeostasis and rebalancing of pathogenic and immunoregulatory networks. At this stage, strategies aimed at limiting antigen-presenting cell activation, restraining Th17-skewed responses, reducing early macrophage–FLS inflammatory coupling, or enhancing regulatory programs mediated by Treg cells and immunomodulatory mesenchymal stem cells may be particularly relevant for remission induction. In the middle stage, once self-amplifying inflammatory loops, synovial hyperplasia, and pathogenic stromal expansion are more firmly established, therapeutic priorities may shift toward interrupting chronic inflammatory amplification, suppressing aberrant FLS proliferation and invasion, weakening macrophage–fibroblast–lymphocyte crosstalk, and preventing continued progression toward structural damage. By contrast, in the late stage, when fibrosis, pannus stabilization, dysregulated bone remodeling, and tissue destruction become increasingly dominant, therapeutic strategies may be less capable of fully restoring immune balance and may instead need to focus more strongly on limiting fibrotic remodeling, restraining invasive pannus behavior, preserving residual joint integrity, and slowing the accumulation of irreversible damage. Thus, the timing of intervention may be as important as the molecular target itself, since the same therapeutic strategy may exert different biological effects depending on the stage of synovial microenvironmental evolution at which it is applied.

The extensive pathway crosstalk detailed above further supports a stage-adapted and network-oriented therapeutic framework. Reciprocal activation between NF-κB and MAPK may stabilize inflammatory amplification once self-sustaining loops have been established, whereas cooperative interactions between JAK-STAT and NF-κB may sustain stromal activation and compromise durable remission. In advanced disease, synergistic activity between TGF-β/Smad and Wnt-related signaling appears to promote fibrosis, pannus stabilization, and progressive loss of tissue plasticity. These observations suggest that multi-target, stage-adapted intervention may be more effective than single-pathway suppression in promoting remission, delaying progression, and reducing irreversible tissue injury.

Despite substantial progress, several challenges remain. The spatiotemporal dynamics of synovial cellular interactions are still incompletely understood, particularly with regard to how distinct fibroblast, macrophage, adipocyte-associated, and vascular-associated subsets contribute to transitions between inflammatory activity, remission, and structural remodeling. In addition, although major signaling pathways have been well characterized, the molecular basis of pathway crosstalk, compensatory reprogramming, and network resilience remains insufficiently resolved. The biological mechanisms underlying remission failure and relapse also require deeper investigation, especially in patients who achieve partial suppression of inflammation without stable restoration of local immune balance. Addressing these questions will require integrated multi-omics approaches, spatially resolved functional analyses, and closer correlation between synovial molecular phenotypes and longitudinal clinical outcomes.

Overall, RA may be more appropriately understood as a disease of dynamic microenvironmental imbalance in which pathogenic cellular circuits, regulatory programs, and signaling networks evolve across disease stages and jointly determine inflammatory activity, remission potential, and structural outcome. Future research should therefore move beyond simple identification of pathogenic factors and instead focus on defining the stage-specific cellular programs and interaction networks that determine whether the synovial microenvironment remains locked in persistent inflammatory activity, rebalances toward remission, or progresses into fibrosis and irreversible structural damage. Such efforts may provide a stronger conceptual basis for mechanism-informed, stage-adapted precision therapies aimed not only at suppressing inflammation, but also at restoring synovial immune homeostasis, improving remission durability, delaying structural progression, and reducing the transition from reversible inflammation to irreversible tissue injury.

## Conclusion

Within the RA synovial microenvironment, diverse stromal, vascular, metabolic, and immune cell populations form a dynamic interaction network that drives disease progression along a pathological continuum from early autoimmune initiation, through middle-stage inflammatory amplification and synovial hyperplasia, to late fibrosis, pannus formation, dysregulated bone remodeling, and irreversible cartilage and bone destruction. Accordingly, RA progression should be understood not simply as the consequence of isolated pathogenic mediators, but as the result of sustained imbalance between pathogenic cellular circuits and immunoregulatory programs within the synovial microenvironment. This imbalance not only promotes structural progression, but also helps determine whether the joint remains in a state of persistent inflammatory activity, achieves only unstable disease control, or re-enters a remission-associated and relatively rebalanced state.

Importantly, the biological and therapeutic implications of these findings are stage-dependent. During the early stage, when key cellular subsets and interaction programs may still retain substantial plasticity, modulation of pathogenic crosstalk and restoration of local immune homeostasis may offer an important opportunity to promote remission. Even in the middle stage, targeted disruption of self-amplifying inflammatory loops and stromal pathogenic programs may still help delay progression and reduce irreversible tissue injury. By contrast, in late-stage disease, when fibrosis, pannus stabilization, and structural destruction become dominant, therapeutic priorities may need to shift toward limiting maladaptive remodeling and preserving residual joint integrity.

Overall, the central conclusion of this review is that RA synovial pathology should be interpreted as a stage-dependent and network-driven process rather than as the consequence of isolated inflammatory mediators or single-cell abnormalities. A deeper understanding of the stage-specific cellular programs, interaction networks, and signaling pathway dynamics that govern inflammatory activity, remission failure, and structural progression may provide a stronger conceptual basis for mechanism-informed precision therapies in RA. This perspective also helps explain why therapeutic strategies targeting a single pathway may be insufficient in some patients and supports the need for stage-adapted, network-oriented interventions that restrain pathogenic cellular circuits, preserve residual tissue plasticity, restore immunoregulatory balance, promote durable remission, and prevent the transition from reversible inflammation to irreversible structural joint damage.

## Data Availability

Not applicable.

## Abbreviations

Akt: Protein kinase B
Ang II: Angiotensin II
Ang-1: Angiopoietin-1
anti-CCP: Anti-cyclic citrullinated peptide
AP-1: Activator protein-1
BAFF: B-cell activating factor
C3aR: C3a receptor
CAP1: Adenylate cyclase-associated protein 1
CCL2: c-c motif chemokine ligand 2
CD40L: CD40 ligand
cell-ECM: Cell-extracellular matrix
ChemR23: Chemerin chemokine-like receptor 1
CIA: Collagen-induced arthritis
c-Rel: REL proto-oncogene
CXCL12: c-x-c motif chemokine ligand 12
CXCL13: c-x-c motif chemokine ligand 13
CXCL8: c-x-c motif chemokine ligand 8
CXCR4: c-x-c chemokine receptor type 4
DCs: Dendritic cells
ECM: Extracellular matrix
Elk-1: E26 transformation-specific like-1 protein
ELS: Ectopic lymphoid structures
ERK: Extracellular signal-regulated kinase
EVs: Extracellular vesicles
FAK: Focal adhesion kinase
FGF-2: Fibroblast growth factor 2
FGFRs: Fibroblast growth factor receptors
FLSs: Fibroblast-like synoviocytes
FOXP3: Forkhead box protein P3
FZD: Frizzled receptor
GM-CSF: Granulocyte-macrophage colony-stimulating factor
gp130: Glycoprotein 130
HA: Hyaluronic acid
HGF: Hepatocyte growth factor
ICAM-1: Intercellular adhesion molecule 1
IDO: Indoleamine 2,3-dioxygenase
IFN-β: Interferon-beta
IKK: IκB kinase
IL-12: Interleukin-12
IL-17: Interleukin-17
IL-17A: Interleukin-17 A
IL-17F: Interleukin-17 F
IL-17RA/RC: Interleukin-17 receptor A/C
IL-1R: Interleukin-1 receptor
IL-1β: Interleukin-1 beta
IL-22: Interleukin-22
IL-23: Interleukin-23
IL-6: Interleukin-6
IL-6R: Interleukin-6 receptor
IL-8: Interleukin-8
IPFP: Infrapatellar fat pad
IκB: Inhibitor of kappa B
IκBα: Inhibitor of nuclear factor kappa-B alpha
JAK-STAT: Janus kinase/signal transducer and activator of transcription
JAK1: Janus kinase 1
JAK2: Janus kinase 2
JAK3: Janus kinase 3
JNK: c-Jun N-terminal kinase
LFA-1: Lymphocyte function-associated antigen-1
LRP5/6: Low-density lipoprotein receptor-related protein 5/6
Mac-1: Macrophage antigen-1
MAPK: Mitogen-activated protein kinase
MCP-1: Monocyte chemoattractant protein-1
MEK: Mitogen-activated protein kinase kinase
MHC class II: Major histocompatibility complex class II
miRNAs: MicroRNAs
MLSs: Macrophage-like synoviocytes
MMPs: Matrix metalloproteinases
mRNAs: Messenger RNAs
MSCs: Mesenchymal stem cells
NF-κB: Nuclear factor kappa-light-chain-enhancer of activated B cells
Ob-R: Leptin receptor
p50: Nuclear factor kappa B subunit 1, processed from precursor p105
p52: Nuclear factor kappa B subunit 2, processed from precursor p100
p65: RELA proto-oncogene
PD-1: Programmed cell death protein 1
PDGF: Platelet-derived growth factor
PD-L1: Programmed death-ligand 1
PGE2: Prostaglandin E2
PI3K: Phosphoinositide 3-kinase
PPARγ: Peroxisome proliferator-activated receptor gamma
RA: Rheumatoid arthritis
Raf: Rapidly accelerated fibrosarcoma
RAS: Renin-angiotensin system
RF: Rheumatoid factor
RUNX2: Runt-related transcription factor 2
SASP: Senescence-associated secretory phenotype
SFs: Synovial fibroblasts
Smad: Mothers against decapentaplegic homolog
Smad2/3: Mothers against decapentaplegic homolog 2 and 3
Smad4: Mothers against decapentaplegic homolog 4
Smad6: Mothers against decapentaplegic homolog 6
Smad7: Mothers against decapentaplegic homolog 7
STAT1: Signal transducer and activator of transcription 1
STAT2: Signal transducer and activator of transcription 2
STAT3: Signal transducer and activator of transcription 3
STAT4: Signal transducer and activator of transcription 4
STAT5: Signal transducer and activator of transcription 5, including subtypes 5a and 5b
STAT6: Signal transducer and activator of transcription 6
STMs: Synovial tissue macrophages
TCF/LEF: T-cell factor/lymphoid enhancer-binding factor
TGF-β: Transforming growth factor-beta
Th17: T helper 17
TLR4: Toll-like receptor 4
TNF-α: Tumor necrosis factor-alpha
TNFR: Tumor necrosis factor receptor
Tph cells: Peripheral helper T cells
Treg: Regulatory T cells
TSG-6: Tumor necrosis factor-stimulated gene 6
TYK2: Tyrosine kinase 2
TβR: TGF-β receptor
TβRI: TGF-β type I receptor
TβRII: TGF-β type II receptor
VCAM-1: Vascular cell adhesion molecule 1
VECs: Vascular endothelial cells
VEGF: Vascular endothelial growth factor
VEGFR: Vascular endothelial growth factor receptor
VLA-4: Very late antigen-4
VSMCs: Vascular smooth muscle cells
Wnt3a: Wnt family member 3 A
α4β1: Integrin α4β1, also known as very late antigen-4
β-cat: β-catenin
DVL: Dishevelled
FcγR: Fc gamma receptor
FN: Fibronectin
ICs: Immune complexes
iNOS: Inducible nitric oxide synthase
miR-155: MicroRNA-155
mTOR: Mechanistic target of rapamycin
Mφ: Macrophage
TFs: Transcription factors
VTN: Vitronectin
